# Design of Glycoengineered IL-4 Antagonists Employing Chemical and Biosynthetic Glycosylation

**DOI:** 10.1021/acsomega.3c00726

**Published:** 2023-07-05

**Authors:** Sarah Thomas, Juliane E. Fiebig, Eva-Maria Kuhn, Dominik S. Mayer, Sebastian Filbeck, Werner Schmitz, Markus Krischke, Roswitha Gropp, Thomas D. Mueller

**Affiliations:** † Department of Molecular Plant Physiology and Biophysics, Julius-von-Sachs Institute of the University Wuerzburg, Julius-von-Sachs Platz 2, D-97082 Wuerzburg, Germany; ‡ Department of Biochemistry and Molecular Biology, Biocenter of the University Wuerzburg, Am Hubland, D-97074 Wuerzburg, Germany; § Department of Pharmaceutical Biology, Julius-von-Sachs Institute of the University Wuerzburg, Julius-von-Sachs Platz 2, D-97082 Wuerzburg, Germany; ∥ Department of General- Visceral-, Vascular- and Transplantation Surgery, Hospital of the LMU, Nussbaumstr. 20, 80336 Munich, Germany

## Abstract

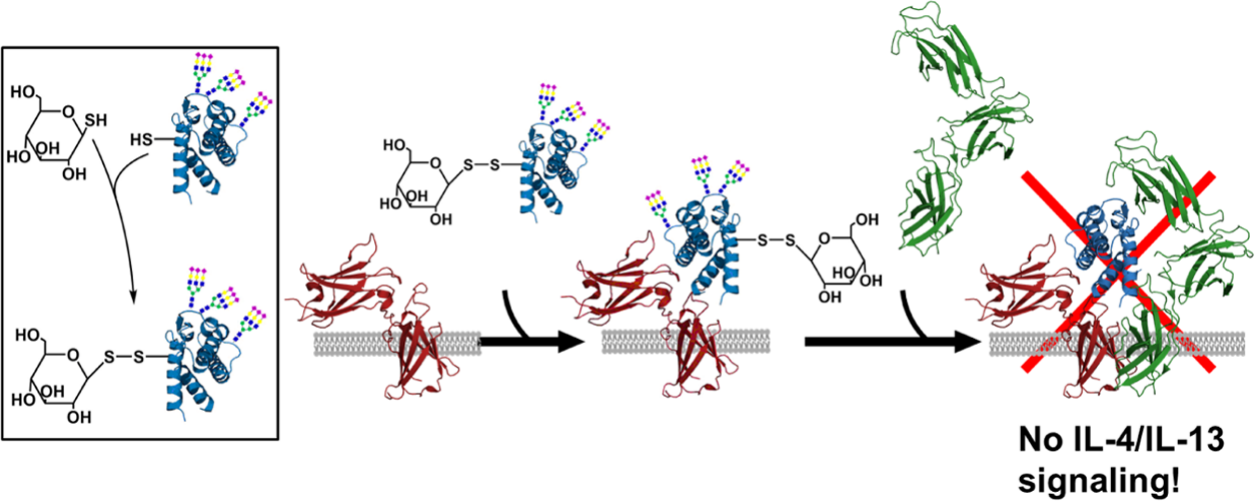

Interleukin-4 (IL-4) plays a key role in atopic diseases. It coordinates T-helper cell differentiation to subtype 2, thereby directing defense toward humoral immunity. Together with Interleukin-13, IL-4 further induces immunoglobulin class switch to IgE. Antibodies of this type activate mast cells and basophilic and eosinophilic granulocytes, which release pro-inflammatory mediators accounting for the typical symptoms of atopic diseases. IL-4 and IL-13 are thus major targets for pharmaceutical intervention strategies to treat atopic diseases. Besides neutralizing antibodies against IL-4, IL-13, or its receptors, IL-4 antagonists can present valuable alternatives. Pitrakinra, an *Escherichia coli*-derived IL-4 antagonist, has been evaluated in clinical trials for asthma treatment in the past; however, deficits such as short serum lifetime and potential immunogenicity among others stopped further development. To overcome such deficits, PEGylation of therapeutically important proteins has been used to increase the lifetime and proteolytic stability. As an alternative, glycoengineering is an emerging strategy used to improve pharmacokinetics of protein therapeutics. In this study, we have established different strategies to attach glycan moieties to defined positions in IL-4. Different chemical attachment strategies employing thiol chemistry were used to attach a glucose molecule at amino acid position 121, thereby converting IL-4 into a highly effective antagonist. To enhance the proteolytic stability of this IL-4 antagonist, additional glycan structures were introduced by glycoengineering utilizing eucaryotic expression. IL-4 antagonists with a combination of chemical and biosynthetic glycoengineering could be useful as therapeutic alternatives to IL-4 neutralizing antibodies already used to treat atopic diseases.

## Introduction

Interleukin (IL)-4 is produced by hematopoietic cells and exerts multiple effects on the immune system. One major function is the regulation of T-helper cell development, where it triggers the differentiation of naïve CD4^+^ T-helper cells (Th0) into subtype 2 T-helper cells (Th2).^1−4^ Mature Th2-cells secrete a specific subset of cytokines, including IL-4, IL-5, and IL-13 that mediate an inflammatory immune response against extracellular pathogens.^5^ By secreting IL-4, Th2-cells suppress the development of the Th1 phenotype thereby maintaining Th2 cell activity.^6,7^ Physiological effects of IL-4 and IL-13 partially overlap. Both cytokines induce the upregulation of vascular cell adhesion molecule-1 (VCAM1) and thus facilitate leukocyte migration to the site of infection.^8,9^ Most importantly, IL-4 and IL-13 drive immunoglobulin class switching to immunoglobulin E (IgE) in B-cells.^10,11^ In addition, IL-4 and IL-13 upregulate Fcε-receptors on *e.g.*, mast cells and B-cells.^12−15^ IL-4/IL-13-mediated IgE sensitization is of great pathophysiological significance, as IgE plays a major role in atopic diseases, such as asthma, atopic dermatitis, and allergic rhinitis.^16^ The recognition of allergens through the IgE/Fcε complex on mast cells and basophilic or eosinophilic granulocytes stimulates their degranulation leading to the release of pro-inflammatory mediators, like the anticoagulant heparin and the vasodilator histamine. Prostaglandins, leukotrienes, and proteases are also released, facilitating local inflammation by causing muscle constriction and tissue remodeling.^17^ Their crucial role in the pathophysiology of atopic diseases has made both cytokines, IL-4 and IL-13, key targets for novel therapeutic approaches to fight allergies and asthma.^18^

IL-4 and IL-13 share a set of transmembrane receptors, which upon ligand-mediated activation induce the JAK/STAT signaling cascade.^19^ IL-4 can form two heterodimeric receptor assemblies, termed interleukin-4 type I and type II receptors. In a sequential process, IL-4 first binds to IL-4 receptor α (IL-4Rα) (CD124) and this membrane-located complex then recruits one of the two other receptors, either the so-called common γ chain (short: γc; CD132) forming the IL-4 type I receptor, or IL-13Rα1 (CD213a1) to yield the IL-4 type II receptor.^20^ The type I receptor is activated exclusively by IL-4, although γc itself is shared with other cytokines IL-2, IL-7, IL-9, IL-15, and IL-21.^21^ The IL-4 type II receptor is used by IL-4 and IL-13 and hence both cytokines assemble and activate the same receptor, explaining their overlapping functions. This receptor sharing allows simultaneous inhibition of both cytokines by targeting the common receptor IL-4Rα.^22^ As a classical approach, anti-IL-4Rα antibodies, AMG317 (Amgen) and Dupilumab (Regeneron/Sanofi), have been developed which simultaneously neutralize IL-4 and IL-13 signaling.^23^ Dupilumab was approved for treatment of atopic dermatitis, thereby confirming IL-4Rα as a therapeutic target for atopic diseases.^24,25^ A second, non-antibody-based strategy to inhibit IL-4 and IL-13 has been implemented employing a mechanistically similar approach to that for Dupilumab. From mutagenesis two positions in IL-4 were shown to convert IL-4 into an effective antagonist that binds IL-4Rα with wild-type-like affinity, but is incapable of activating either the type I or type II receptor as it cannot bind γc or IL-13Rα1.^26−28^ This IL-4 variant harboring mutations R121D and Y124D was termed Pitrakinra. It was tested for application in allergic asthma until clinical phase trial 2a, where it failed to meet endpoint criteria. Further developments of Pitrakinra were stopped, although positive therapeutic effects, *e.g.*, fewer allergen-induced exacerbations, could be observed.^29^ A major disadvantage seemed its very short half-life *in vivo* due to the small size and lack of glycosylation, resulting from its production in prokaryotic expression systems.^30^ To overcome these problems short PEGylation was tested. However, even though PEG coupling was done site-specific, the modification significantly impaired inhibitor efficacy.^31^ Here, we present an alternative to Pitrakinra, which evades its inherent disadvantages using a novel approach to simultaneously implement IL-4 antagonism and improve its pharmacological properties.

## Results

### New Strategy to Generate an IL-4 Antagonist – Glycan-Mediated Steric Hindrance

Pitrakinra exerts its antagonistic effect by introducing an electrostatic mismatch in the binding epitopes to γc and IL-13Rα1.^32^ We hypothesized that implementing steric hindrance instead might yield a superior antagonist, as binding to γc or IL-13Rα1 can be more effectively blocked. Duppatla et al. coupled thiol-reactive, bulky reagents to cysteine residues introduced in IL-4′s binding epitope for γc and IL-13Rα1 to induce steric hindrance.^33,34^ They found that the site-specific modification of a single IL-4 residue, i.e., Arg121 is sufficient to disrupt binding to γc and IL-13Rα1. However, the authors used either maleimide-containing molecules, *e.g.*, N-ethylmaleimide, that might be immunogenic when presented by IL-4 in a hapten-like manner or large polymers such as PEG, which are heterogeneous and can negatively influence antagonist efficacy due to their sheer size as seen for PEGylated Pitrakinra.^31^ In addition, PEG shows poor degradability leading to accumulation in the liver and in some, albeit in rare cases anti-PEG antibodies were detected that would invalidate the therapeutic.^35^ For the development of an improved IL-4 antagonist we therefore wanted to use modifications to introduce steric hindrance, which are naturally present on the human IL-4 protein surface. Glycosylation is a native modification found in many secreted proteins, which can decrease immunogenicity and prolong the serum half-life.^36,37^

We first tested whether an N-glycan moiety can be installed at position 121 by mutating the target site to encode an N-glycosylation motif, i.e., Asn121-Glu122-Ser123. To facilitate analysis, the native N-glycosylation site in IL-4 at Asn38 was silenced,^38^ i.e., via mutation N38Q, thereby generating a variant with a single potential N-glycosylation site. Expression in HEK293 cells, however, revealed that the engineered site was not modified (see Figure S1). This suggests that introducing a glycan moiety at position 121 requires a post-translational chemical procedure and cannot be achieved by biosynthetic means.

### Coupling of a Carbohydrate to Position 121 via a Bifunctional Chemical Crosslinker

For chemical coupling, we introduced a thiol group as the reactive site for several reasons (see also ref (39)). As IL-4 does not contain any unpaired cysteine,^38^ the introduced thiol group will allow site-specific coupling. Second, thiol coupling can be performed under near-physiological conditions. Conjugation occurs fast and unspecific reaction with other polar groups in proteins is not observed. We used prokaryotic expression to produce IL-4 in insoluble form in so-called inclusion bodies. After extraction, an oxidative *in vitro* refolding is performed to obtain IL-4 in its native fold with all three native disulfide bonds formed. All variants contained the mutation F82D as this mutation enhances the affinity for IL-4Rα about 3-fold,^40^ which enables the IL-4 antagonist to more effectively outcompete endogenous wild-type IL-4. After expression in *Escherichia coli*, the IL-4 F82D R121C protein was extracted, and *in vitro* refolding was performed using a glutathione-based redox couple to facilitate disulfide formation. Under this condition, the unpaired cysteine (Cys121) formed a mixed disulfide with glutathione. While this protects from non-specific side reactions, subsequent removal of this “protection” group is required before carbohydrate coupling can proceed. Removal of this glutathionyl group by chemical reduction failed, as all reducing agents tested also simultaneously cleaved IL-4’s native disulfide bonds. This led to denaturation and irreversible precipitation of the protein. For specific removal, Duppatla et al. developed an enzymatic procedure utilizing *E. coli* GSH-disulfide oxidoreductase glutaredoxin-1 (grxA).^33^ In the presence of reduced glutathione (GSH) the enzyme preferentially reduces the mixed glutathione-disulfide. It therefore generates a free thiol group at the unpaired cysteine and oxidized glutathione (GSSG). As the latter inhibits glutaredoxin, GSSG must be recycled using glutathione reductase and NADPH. Although glutaredoxin-1 preferentially cleaves GSH-Cys mixed disulfides, it can also reduce regular cystine disulfide bonds albeit at a slower rate. Hence reaction times and GSH concentration for enzymatic removal of glutathione from mixed disulfides had to be optimized. For IL-4 F82D R121C, a reaction time of 2.5 min was sufficient to achieve nearly complete conversion of Cys121 into its non-conjugated, free thiol form, longer reaction times led to enhanced cleavage of IL-4’s native disulfide bonds, shorter reaction times did not sufficiently cleave the GSH-Cys mixed disulfide bond (Figure S2A,B). The protein could be recovered in high purity with about 50–60% yield. Subsequent oxidation of the thiol group was prevented by acidifying the protein solution to pH ≤ 6.

**Figure 1:**
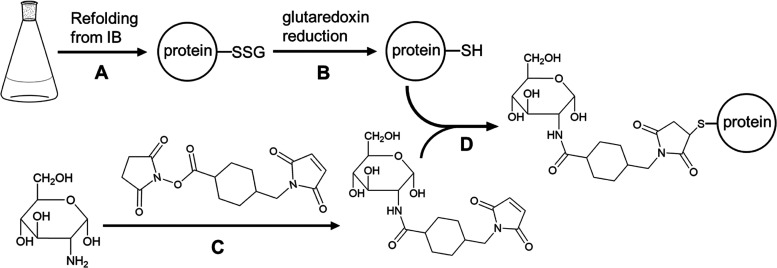
Conjugation of amino sugars to the thiol group in IL-4 F82D R121C employing the bifunctional crosslinker SMCC. (A) Bacterial expression and oxidative refolding of IL-4 variants harboring an unpaired cysteine in the presence of a glutathione redox couple. (B) Enzymatic deglutathionylation of the mixed glutathione-disulfide bond using the glutaredoxin system yielding a free thiol group at the engineered cysteine. (C) Reaction of the bifunctional crosslinker SMCC with glucosamine. (D) Site-specific conjugation of the SMCC-glucosamine conjugate to the thiol group of the engineered cysteine.

As the composition and architecture of the carbohydrate to be coupled affect parameters, such as immunogenicity, stability, and serum half-life, we first developed a coupling scheme for complex glycans isolated from natural protein sources. The latter could be obtained by enzymatic hydrolysis using endoglycosidase PNGase F, which yields complex glycans with a 1-amino-β-GlcNAc form at the reducing end. Coupling of such an amino-saccharide to a thiol requires a (hetero)-bifunctional chemical crosslinker equipped with a thiol-reactive as well as an amino-reactive group (Figure 1). The reaction can be performed under near-physiological conditions but requires a defined sequential procedure due to other reactive amino groups (*e.g.*, lysine) in the protein, which would form unwanted conjugates. Succinimidyl 4-(*N*-maleimidomethyl) cyclohexane-1-carboxylate (SMCC) was chosen as the crosslinker as the cyclohexane ring restrains the conformational freedom required to establish steric hindrance for conversion of IL-4 into an antagonist.

Since the availability of complex glycans is limited, a proof-of-concept study was performed using 2-glucosamine-HCl, which carries an amino group at C-atom 2 (Table S1). First, the glucosamine–SMCC conjugate was prepared to prevent conjugation of the SMCC NHS-activated carboxylate group to amino groups in IL-4. SMCC was coupled to 2-glucosamine-HCl in a molar ratio of 1:4. Mass spectrometry analysis (for experimental procedure see the Supporting Information), however, revealed a low conversion yield, demanding efficient purification of the conjugate to avoid the presence of amino-reactive, non-coupled SMCC in the subsequent reaction with IL-4. Reversed-phase chromatography was used due to the high hydrophobicity of SMCC, which decreased upon coupling to glucosamine. Both products could be separated with SMCC-glucosamine elution at 55% acetonitrile and non-reacted SMCC at ≥90% acetonitrile (Figure S3). Using the above-described setup the overall yield of the conjugate was about 5% only based on SMCC as an educt. The low coupling yield might be partly related due to the low pH of the coupling reaction since dissolving glucosamine-HCl in phosphate buffer at the given concentrations resulted in a shift in the pH of the reaction mixture to 6.5, possibly too low for efficient coupling of the amine to the N-hydroxysuccinimide activated carboxylate group in SMCC. We therefore, tried to optimize the first coupling step, however, reaction conditions for this coupling reaction with SMCC are rather limited. While a more basic pH can increase the efficiency of the amino coupling reaction through the activated NHS ester group, a pH value of the reaction mixture above 7.5 will lead to unspecific (competitive) coupling of amines to the maleimide group.^41^ In addition, at basic pH maleimide groups suffer fast hydrolysis, which would impede the second coupling of the glucose-crosslinker adduct to IL-4.^41^ To limit hydrolysis, we have chosen SMCC as a bifunctional crosslinker, in which the cyclohexane ring stabilizes the adjacent maleimide group.^42^ This, however, strongly decreases the solubility of the crosslinker as well as of the glucosamine-SMMC product hampering subsequent purification efforts. Alternative coupling conditions were tested employing a pH value of 7.2 (measured in the reaction mixture) for the first coupling step and testing dimethyl sulfoxide instead of acetonitrile as the solvent for SMCC. Despite these efforts coupling of glucosamine to SMCC did not exceed a yield of more than 25–30%. An alternative synthesis route, *e.g.*, first coupling SMCC to the IL-4 protein is also not possible though. Due to the large number of lysine residues in the protein, the conjugation of the SMCC crosslinker to IL-4 would result in significant coupling of SMCC to these amine groups instead of the desired conjugation to the introduced cysteine residue. The highly purified glucosamine-SMCC conjugate derived from the above-described reaction was then reacted for 2 h at room temperature (RT) with enzymatically deglutathionylated IL-4 F82D R121C using a molar ratio of 100:1 and the reaction setup was purified by reversed-phase high-performance liquid chromatography (RP-HPLC) (Figure S4). Mass spectrometry confirmed that this procedure afforded the desired protein glycoconjugate and coupling to Cys121 occurred specifically (Table S2 and Figure S5). While the second coupling step could be performed with a high conversion rate, the overall strategy is seriously handicapped by the low yield for the first SMCC-carbohydrate coupling reaction and hence this strategy was not developed any further.

### Linker-Free Coupling of Thiol-Carbohydrates Using Phenylselenyl Bromide Activation

**Figure 2:**
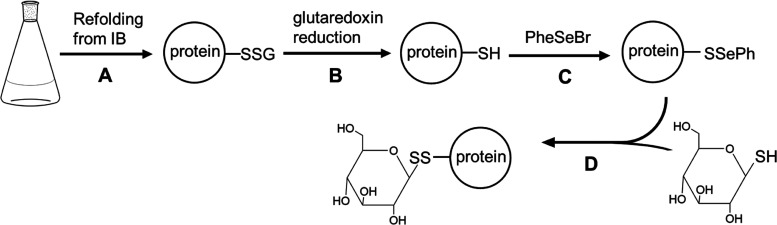
Conjugation of thiol-carbohydrates to IL-4 F82D R121C using phenylselenyl bromide activation. (A) Bacterial expression and oxidative refolding of IL-4 variants harboring unpaired cysteine in the presence of a glutathione redox couple. (B) Enzymatic reduction of the glutathione-mixed disulfide using the glutaredoxin system yielding engineered cysteine with a free thiol. (C) Activation of the free thiol of the engineered cysteine with phenylselenyl bromide to yield mixed phenylselenylsulfide. (D) Conjugation of the thiol-carbohydrate, yielding a disulfide-linked glycoprotein conjugate.

Hence, a different approach was developed, which allows direct/linker-free conjugation of a carbohydrate moiety to engineered cysteine residues. A glycoengineering strategy, established by Gamblin and colleagues termed Glyco-SeS,^43^ utilizes thiol-functionalized carbohydrates (see Table S1) for linker-free conjugation. Reaction with a phenylselenylating reagent converts the free sulfhydryl group into a mixed phenylselenylsulfide.^43−45^ Because of the electrophilic nature of sulfur in this S–Se bond, it readily reacts with thiol-containing carbohydrates to form disulfide-linked protein–carbohydrate conjugates (Figure 2).

Alternatively, thio-carbohydrates can be activated with phenylselenyl bromide, which is then coupled to a free thiol in the protein. The approach is also applicable to glycans isolated from natural sources, although the polysaccharide has to be thiol-functionalized prior to coupling, *e.g.*, through reaction with Lawesson′s reagent.^44^

To test this approach commercially available 1-thio-β-d-glucose sodium salt and the acetylated derivative 1-thio-β-d-glucose tetraacetate were used as model substrates. Deglutathionylated IL-4 F82D R121C was treated with a 40-fold molar excess of phenylselenyl bromide for 1 h. After the removal of excess phenylselenyl bromide by size exclusion, the “thiol-activated” protein was reacted with an 80-fold molar excess of thio-glucose for 40 min at RT. Unfortunately, no full conversion could be achieved and mass spectrometry revealed about 10% non-modified IL-4 F82D R121C. Because non-conjugated IL-4 F82D R121C exerts agonistic activity,^34^ it must be removed. Non-conjugated IL-4 F82D R121C could be efficiently removed by covalent binding to iodoacetyl-activated agarose due to its reactive thiol group (see the Supporting Information). Thereby, pure IL-4 F82D R121C-SS-(4ac)Glc and IL-4 F82D R121C-SS-Glc were obtained (Figure S6). Thus, the Glyco-SeS method can be used for crosslinker-free preparation of disulfide-linked IL-4 glycoproteins. However, a large excess of thio-carbohydrates is needed, conversion was nonetheless incomplete, and removal of non-modified protein was necessary. In addition, enzymatic deglutathionylation of the engineered cysteine is still a prerequisite, which potentially limits large-scale synthesis.

### Direct Coupling of Thiol-Carbohydrates during Refolding of IL-4

**Figure 3:**
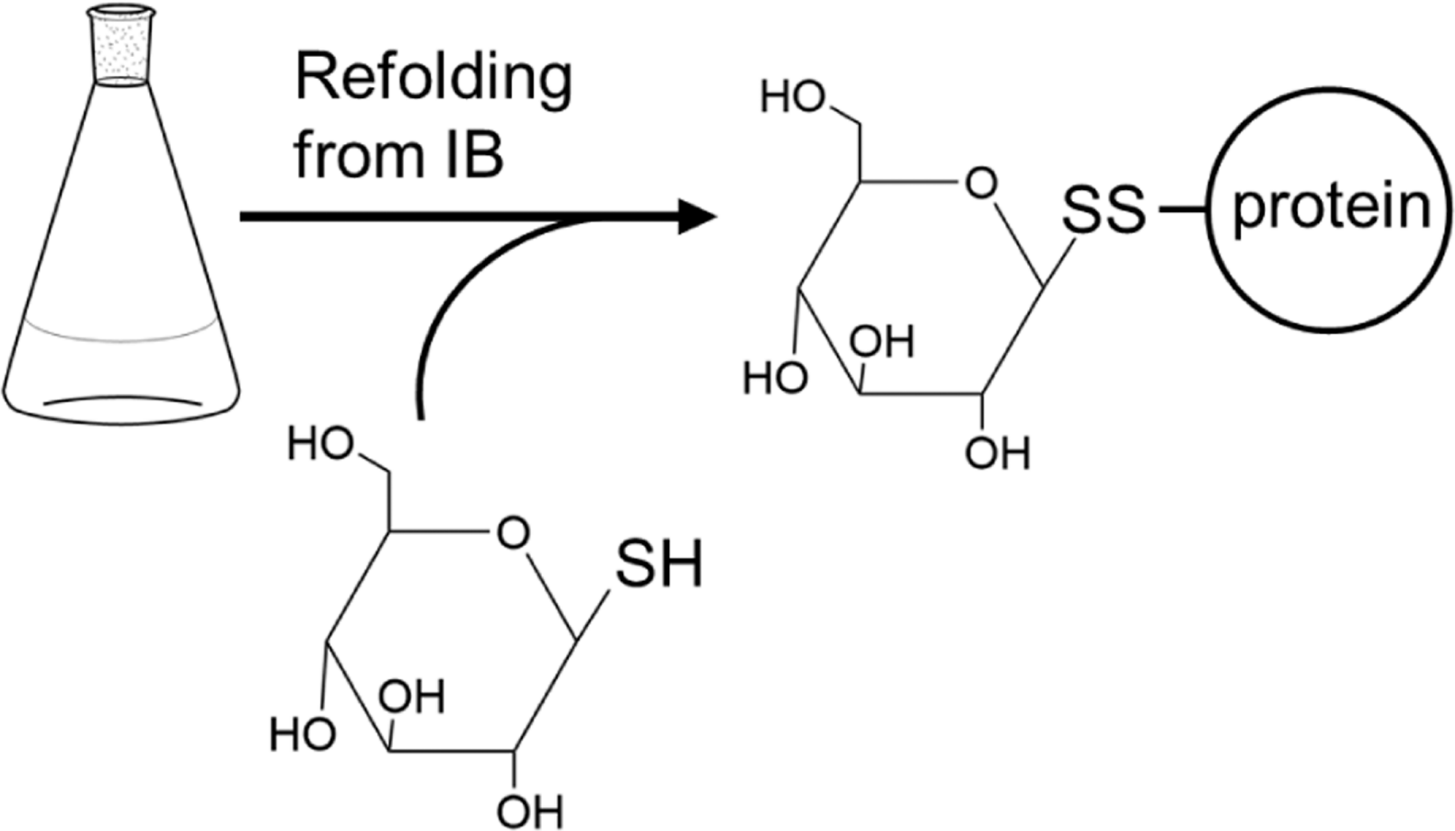
Conjugation of thiol-carbohydrates to IL-4 F82D R121C during refolding. Bacterial expression and oxidative refolding of IL-4 cysteine variants in the presence of thiol-carbohydrate.

Refolding of bacteria-derived IL-4 is done with a glutathione GSH/GSSG redox couple to facilitate disulfide bond formation and to improve the yield of the correctly folded protein. We thus wondered whether disulfide-linked IL-4 glycoconjugates could be generated by adding thio-carbohydrates instead of glutathione to refolding (Figure 3).

IL-4 F82D R121C was extracted from inclusion bodies and the protein solution was added to renaturation buffer to yield a protein concentration of 50 μg/mL. Refolding was performed in the presence of 1 M arginine and 1 mM 1-β thio-glucose (or its tetraacetate derivative). Although no oxidizing GSSG was present, the refolding yield was identical to conditions containing a glutathione redox couple (about 10 mg IL-4/g *E. coli* biomass). Mass spectrometry verified that all three native disulfide bonds were correctly formed and that Cys121 was disulfide-bonded to glucose (or its tetraacetate derivative) with no non-conjugated protein present (Figure S5).

The scheme was then tested for whether it is limited to single-site glycoconjugation or whether multiple thio-saccharides can be coupled to IL-4 variants carrying several unpaired cysteines. For proof of principle, variant F82D R121C N38C was generated containing a second unpaired cysteine at position 38. Refolding was performed as above, delivering the same yield as for the IL-4 single-site conjugate F82D R121C. Mass spectrometry identified one major species with the molecular weight of IL-4 F82D R121C N38C comprising three disulfide bonds and with both engineered cysteines conjugated to thio-glucose (or its tetraacetate derivative) (Figure S5). In contrast to IL-4 F82D R121C, also non-modified and IL-4 F82D R121C N38C, conjugated only at one site, were obtained, indicating that conjugation of thio-saccharides to several sites likely requires adjustment of reaction conditions for full conversion (Figure S5). This hypothesis is corroborated by the finding that the bulkier tetraacetate thio-glucose showed a lower coupling rate than the smaller thio-glucose.

### Glycoengineered IL-4 F82D R121C Variants Exhibit High-Affinity for IL-4Rα and act as Highly Effective IL-4 Antagonists in Cell-Based Assays

To test whether thio-saccharide coupling to IL-4 R121C affects binding to the receptor IL-4Rα, which would deteriorate antagonist efficacy, surface plasmon resonance (SPR) (see the Supporting Information) was used to determine affinities of glycoengineered IL-4 F82D R121C analogues for IL-4Rα. Analysis of the SPR sensograms (Figure S7) yielded an apparent affinity of 183±42 pM for wild-type IL-4 (non-modified and not containing the IL-4Rα affinity-enhancing mutation F82D), consistent with previous reports.^34,40,46^ IL-4 F82D R121C variants conjugated to different carbohydrate moieties similarly exhibited high-affinity binding to IL-4Rα, which exceeded those of wild-type IL-4 and Pitrakinra by about 9 to 10-fold (Table 1).

**Table 1:** SPR Analysis of IL-4 Variants and Glycoconjugates^a^

IL-4 variant	*k*_off_ × 10^–4^ [s^–1^]	*k*_on_ × 10^6^ [M^–1^ s^–1^]	*K*_D_ [pM]
WT	9.4 ± 0.3	5.3 ± 1.5	183 ± 42
F82D	1.4 ± 0.1	9.5 ± 0.4	14.9 ± 2.0
F82D N38C	1.6 ± 0.2	13.2 ± 0.7	12.3 ± 2.0
F82D N38C-Glc	2.9 ± 1.6	15.5 ± 2.9	18.7 ± 1.0
F82D R121C	1.2 ± 0.3	8.7 ± 0.2	14.1 ± 1.6
F82D R121C-SMCC-GlcN	4.2 ± 0.6	4.6 ± 0.6	93.4 ± 26
F82D R121C-4acGlc	2.6 ± 0.4	7.3 ± 0.8	35.2 ± 3.7
F82D R121C-Glc	1.2 ± 1.0	7.0 ± 6.0	16.6 ± 0.4
F82D R121C-Glc*	1.7 ± 0.3	1.4 ± 0.2	120 ± 24
F82D N38C R121C	0.6 ± 0.3	7.4 ± 0.1	8.4 ± 0.5
F82D (Q20N T28N K61N) R121C-Glc*	2.5 ± 0.5	2.2 ± 1.6	161 ± 82
R121D Y124D	9.6 ± 1. 5	6.0 ± 0.5	163 ± 39

a Association (*k*_on_) and dissociation (*k*_off_) rate constants, as well as equilibrium binding constants (*K*_D_ from *K*_D_ = *k*_off_/*k*_on_) from three independent experiments, are shown (mean and standard deviation). IL-4 variants marked with asterisk were expressed in the eucaryotic expression system FreeStyle293 or Expi293 cells and hence, carry N-glycosylation, IL-4 proteins not marked with * were derived from expression in *E. coli*.

**Figure 4:**
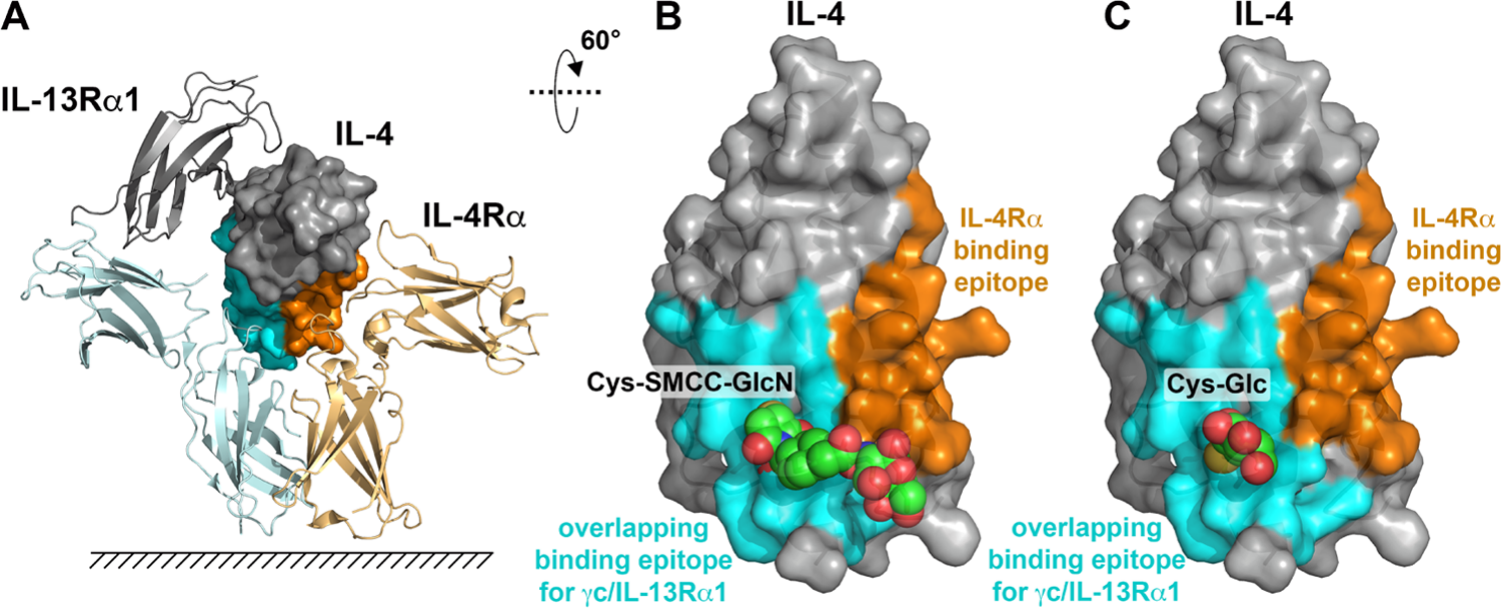
Steric hindrance introduced by site-specific glycoconjugation. (A) Binding sites for the IL-4 receptors IL-4Rα (in light orange) and IL-13Rα1 (in light cyan/gray) were deduced from the crystal structure of the ternary IL-4 type II receptor complex (PDB entry 3BPN(47)) and are marked in orange (IL-4Rα epitope) and cyan (γc/IL-13Rα1 binding site, only the epitope utilized by both receptors is color-coded), respectively. (B) Conjugation of an amine-containing carbohydrate via the bifunctional crosslinker SMCC to Cys121 results in a rather long chain that can possibly extend to the binding site for IL-4Rα, resulting in decreased binding affinity for IL-4Rα, thereby limiting the antagonistic efficacy of such glycoengineered IL-4 variants. (C) Direct coupling of thiol-containing carbohydrates (a glucose moiety is shown) to Cys121 via a disulfide bond limits the steric hindrance to γc/IL-13Rα1 binding.

The increased affinities of the glycoengineered IL-4 variants are mainly attributable to slower dissociation rates (*k*_off_) resulting from the mutation F82D (Table 1). The latter mutation was initially found to turn IL-4 into a superagonist due to its increasing affinity to IL-4Rα by a factor of three- to five-fold.^40^ It was implemented into our glycoengineered IL-4 antagonists to compensate for possible affinity decreases that might come with glycomodifications. For instance, the increase in hydrodynamic radius due to multiple complex N-glycosylation sites in the IL-4 antagonist F82D (Q20N T28N K61N) R121C-Glc leads to a slower association resulting in a lower affinity for IL-4Rα (Table 1, compare to IL-4 F82D). However, equipped with the mutation F82D, the IL-4 antagonist IL-4 F82D (Q20N T28N K61N) R121C-Glc exhibits the same affinity for IL-4Rα as wild-type IL-4 and can thus effectively compete off endogenous IL-4 from the IL-4 receptor. For disulfide-linked glucose- and glucose tetraacetate-modified IL-4 F82D R121C, the SPR analysis revealed affinities of 17 and 35 pM, respectively. The IL-4 conjugate F82D R121C coupled to SMCC-glucosamine exhibited a lower affinity (*K*_D_ about 90 pM). This lower affinity (when compared to disulfide-linked glycoconjugates) points toward steric hindrance of IL-4Rα binding due to the relatively large size of the bifunctional crosslinker, which is absent in IL-4 glycoconjugates in which the carbohydrate is directly coupled via a disulfide bond (see Figure 4).

Biological activities and IL-4 inhibitory capacities of IL-4 F82D R121C glycoconjugates were assessed using two cell-based experiments. HEK-Blue IL-4/IL-13 cells (Invivogen) carry a secreted alkaline phosphatase (SEAP) reporter gene downstream of an IL-4/IL-13 responsive promoter. As HEK-Blue cells only express IL-4Rα and IL-13Rα1, they present a reporter system specific for IL-4 receptor type II signaling. IL-4/IL-13 biological activity can also be quantified using the human erythroleukemic cell line TF-1, which proliferates in response to IL-4 and IL-13.^48^ TF-1 cells express all three receptors IL-4Rα, γc, and IL-13Rα1 (expression was confirmed by qRT-PCR; Figure S8) and can be used to quantify IL-4 receptor type I- and type II-dependent signaling. Both cell lines demonstrated high sensitivity for IL-4 with half-maximal effective concentrations (EC50) of 3.6 ± 1.0 pM for wild-type IL-4 (IL-4 WT) in HEK-Blue cells and 6.7 ± 1.5 pM for wild-type IL-4 in TF-1 cells consistent with the published data.^28,34^

**Figure 5:**
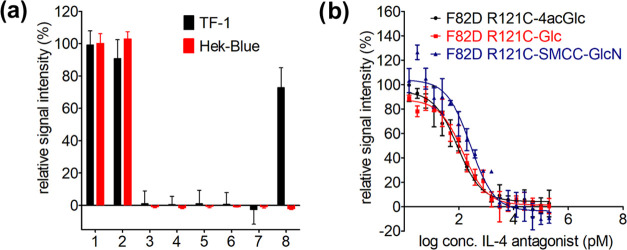
Cell assay analysis of IL-4 F82D R121C glycoconjugates. (a) Biological activity of IL-4 variants and glycoconjugates thereof in HEK-Blue and TF-1 cells. Cells were stimulated with 50 pM of each variant and signal intensity was normalized to wild-type IL-4. Data are from three independent experiments. 1: WT; 2: F82D N38C-Glc; 3: F82D R121C-SMCC-GlcN; 4: F82D R121C-Glc; 5: F82D R121C-4acGlc; 6: F82D R121C-Glc (HEK293); 7: R121D Y124D; 8: F82D R121E. (b) Dose-response curves of IL-4 F82D R121C glycoconjugates in TF-1 cells. Cells were stimulated with a log 2 concentration series of the indicated variants in the presence of 50 pM wild-type IL-4. Signal intensity was normalized to stimulation by 50 pM wild-type IL-4 alone. Data are from three independent experiments.

At a concentration of 50 pM, none of the IL-4 F82D R121C glycoconjugates, irrespective of whether they were conjugated to SMCC-glucosamine or disulfide-linked to glucose or glucose tetraacetate, exhibited any biological activity (<1%) in HEK-Blue or TF-1 cells, similar to the benchmark Pitrakinra (IL-4 R121D Y124D) (Figure 5). This is noteworthy, as for Pitrakinra exchange of Arg121 as well as Tyr124 against aspartate was necessary to shut off signaling through both type I and type II IL-4 receptors. Mutation of only Arg121 in IL-4 R121E blocked signaling via the IL-4, type II receptor, but IL-4 R121E could still efficiently stimulate TF-1 cell proliferation (maximal proliferation was 73 ± 12% compared with wild-type IL-4) consistent with IL-4 R121E being reported as a IL-4 type II receptor-specific antagonist.^49^

This shows that binding of γc and IL-13Rα1 to IL-4 can be efficiently blocked by steric hindrance through a single-site conjugation at position 121 using a monosaccharide, while exchange with other amino acids requires simultaneous mutation at positions 121 and 124 to obtain a full IL-4 antagonist. Hence, steric hindrance as the working principle to generate an IL-4 full antagonist is superior to the electrostatic mismatch strategy used in the design of Pitrakinra.^32^ To test that chemical glycoconjugation during refolding occurs site-specifically to the engineered cysteine residue, the glucose-conjugated IL-4 variants F82D and N38C served as a control. As position 38 presents the natural N-glycosylation site located far outside any receptor epitope, modification with thio-glycans should thus not interfere with IL-4 activity. This conjugate indeed exhibited biological activities identical to wild-type IL-4 (Figure 5), thereby confirming thio-saccharide coupling during refolding occurred specifically on the engineered cysteine and did not alter the native disulfide bonding pattern.

To evaluate antagonistic efficacy of IL-4 F82D R121C glycoconjugates, we determined the half-maximal inhibitory concentration (IC50) by evaluating the competition of IL-4-induced TF-1 cell proliferation (Figure 5), as well as dose-dependent inhibition of IL-4-induced expression of secreted alkaline phosphatase (SEAP) in HEK-Blue cells. For quantitative analysis, the inhibitory constant (*K*_i_) was calculated using the Cheng–Prusoff equation (*K*_i_ = IC_50_/(1 + [IL-4]/EC_50_)).^50^ The inhibitory constant presents a measure of the receptor affinity of an antagonist, i.e., the engineered IL-4 F82D and R121C glycoconjugates for IL-4Rα. The glucose and glucose tetraacetate IL-4 conjugates showed a *K*_i_ of about 20 pM in TF-1 and HEK-Blue cells (determined only for the glucose conjugate), indicating that these novel IL-4 antagonists are 5 times more effective than Pitrakinra (*K*_i_ ≈ 100 pM) (Table 2). With a *K*_i_ of about 50 pM, IL-4-SMCC-GlcN was a slightly less potent antagonist, consistent with its lower IL-4Rα affinity. These data demonstrate that the glycoengineered IL-4 F82D R121C conjugates block IL-4 type I and type II receptors and present highly effective IL-4 antagonists (Figure 5 and Table 2).

**Table 2:** Inhibition of IL-4 Signaling by IL-4 F82D R121C Glycoconjugates^a^

	inhibitory constant (*K*_i_) [pM]	
IL-4 variant	TF-1	HEK-Blue
F82D R121C-SMCC-GlcN	51.2 ± 10.4	ND
F82D R121C-Glc	17.4 ± 6.4	13.1 ± 4.1
F82D R121C-4acGlc	14.6 ± 6.3	ND
R121D Y124D (Pitrakinra)	98.3 ± 21.3	82.4 ± 20.7

a Inhibition constants *K*_i_ were calculated employing the Cheng–Prusoff equation using IC_50_ values of different IL-4 F82D R121C glycoconjugates and the EC_50_ obtained for wild-type IL-4 (TF-1: 6.7 ± 1.5 pM; HEK-Blue: 3.6 ± 1.0 pM). Data were derived from three independent experiments using TF-1 cell proliferation and HEK-Blue SEAP assays. ND = not determined.

### Multi-Glycosylated Variants for Enhanced Protein Stability

**Figure 6:**
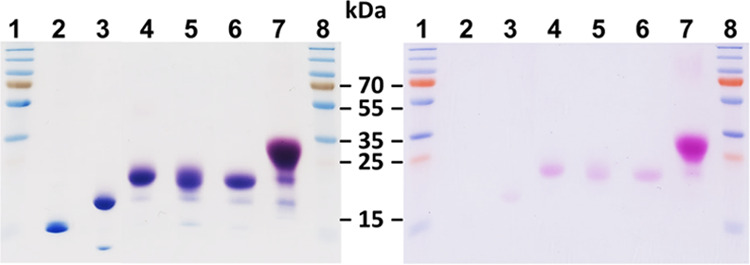
SDS-PAGE analysis of HEK293 cell-derived IL-4 multi-glycosylated variants. An SDS-PAGE analysis was performed under non-reducing conditions and the gel was stained first using acid–Schiff staining (right panel) to highlight glycosylations before Coomassie blue staining was used to detect the protein. 1, 8: molecular weight standard; 2: F82D N38Q; 3: WT; 4: F82D Q20N; 5: F82D T28N; 6: F82D K61N; 7: F82D Q20N T28N K61N.

Pitrakinra exhibited a short serum half-life, estimated to be about 3 h, which severely limited its therapeutic activity *in vivo*.^30^ The small size of 15 kDa and its lack of glycosylation were likely the key determinants, which facilitated fast systemic clearance, *e.g.*, via renal filtration and protein degradation. Accordingly, increasing Pitrakinra′s size through conjugation of a 40 kDa PEG polymer improved serum half-life to about 5 days, but also decreased its efficacy as IL-4 antagonist, as the long PEG moiety critically affected IL-4Rα binding.^31^ As for PEGylated proteins, glycoproteins with complex mammalian-like glycan structures exhibit an increased circulatory lifetime, and are more resistant to proteolysis, and usually have a higher physicochemical stability compared with their non-glycosylated forms.^37^ Since automated chemical synthesis of complex glycans is not yet realized, only a few mostly simpler glycans at a rather high cost are available for chemical glycoengineering. We thus tested whether a multi-glycosylated IL-4 variant containing complex N-glycans can be produced using an eukaryotic expression system and combined with chemical glycosylation of Cys121 to yield an IL-4 antagonist with enhanced pharmacokinetic properties. Several N-glycosylation motifs were introduced in IL-4 F82D by altering the amino acid sequence to N-X-S/T with X not being proline. All sites were chosen to require minimal mutations, ideally a single amino acid exchange, to yield an N-X-S/T degron. Furthermore, only positions outside of secondary structure elements were considered to avoid that these novel sites might not be recognized by glycosyltransferases in the ER. Third, N-glycosylation motifs were introduced at sites that will not interfere with IL-4–IL-4Rα binding, which would impair antagonist efficacy by lowering the binding affinity to the IL-4 high-affinity receptor IL-4Rα. Obeying these requirements three IL-4 single amino acid variants, i.e., Q20N, T28N, and K61N, were produced in HEK293 cells and SDS-PAGE analysis indicated that all three N-glycosylation sites were processed (Figure 6). To confirm that the increase in molecular mass is due to N-glycosylation, the glycoengineered IL-4 variants were subjected to enzymatic hydrolysis employing endoglycosidase PNGase F, which hydrolyzes N-linked oligosaccharides from glycoproteins (Figure S9). From those, we designed an IL-4 multivariant comprising three additional N-glycosylation sites, combining mutations Q20N, T28N, and K61N. SDS-PAGE analysis of IL-4 F82D Q20N T28N K61N revealed an apparent molecular weight of 25–35 kDa (Figure 6), which is about twice the molecular weight of *E. coli*-derived wild-type IL-4/Pitrakinra.

Chemical glycosylation of HEK293 cell-derived IL-4 variants harboring an engineered cysteine required the refolding procedure for modification. Mass spectrometry of such HEK293 cell-derived IL-4 revealed that the unpaired cysteine was conjugated to another cysteine stemming from the cell culture media, which could not be removed by enzymatic deglutathionylation as glutaredoxins are specific for glutathione. Purified IL-4 F82D (Q20N T28N K61N) R121C was therefore first denatured and its cystine residues were reduced using 1 mM DTT. Oxidative refolding was then performed as described above employing rapid dilution with the protein concentration adjusted to 1 mg/mL and in the presence of 1 mM thiol-glucose. Incubation proceeded for 72 h, after which modified IL-4 could be recovered with 30% yield. Mass spectrometry analysis not only confirmed the disulfide formation between Cys121 and thiol-glucose, but also showed that all native disulfide bonds in IL-4 were formed. After purification, only IL-4 protein containing a native disulfide pattern was obtained. SPR analysis was performed to test whether the additional N-glycosylation affects IL-4Rα binding (Figure S7). Chemically glycosylated HEK293 cell-derived IL-4 F82D R121C containing a single N-glycan at position 38 exhibited an affinity of 120±24 pM for IL-4Rα and served as reference. IL-4 variant F82D (Q20N T28N K61N) R121C with N-glycans at positions 20, 28, 38 (native site), and 61 and with Cys121 coupled to thio-glucose bound IL-4Rα with an affinity of 161±82 pM. The 13 times lower affinity compared to bacteria-derived IL-4 F82D R121C-Glc (*K*_D_ 16.6 ± 0.4 pM) is likely due to increased molecular size from the N-glycans, which slows down the association. However, due to the affinity-enhancing mutation F82D,^40^ binding affinities of this multi-glycosylated IL-4 variant are not weaker than wild-type IL-4 (*K*_D_: 183±42 pM) or Pitrakinra (*K*_D_: 163±39 pM) (see also Table 1). Antagonist efficacy was determined from the inhibition of IL-4-mediated TF-1 cell proliferation showing that HEK293 cell-derived IL-4 F82D (Q20N T28N K61N) R121C-Glc exhibits a *K*_i_ value of 184±55 pM (Figure S10) correlating with its *K*_D_ value determined by SPR experiments.

### IL-4 Variants with Additional N-Glycosylation Have Enhanced Proteolytic Stability

Several studies have reported that glycosylation can confer proteolytic stability by masking proteolytic cleavage sites in proteins.^51,52^ We analyzed whether our multi-glycosylated IL-4 variants also show increased proteolytic stability *in vitro*. IL-4 proteins from different expression systems differing in the glycan structure and content were incubated with trypsin as a model protease. Long-term treatment of bacteria-derived IL-4 resulted in complete hydrolysis and thus served as a benchmark. Proteolysis was monitored by SDS-PAGE analysis of samples taken at different incubation times and quantified from Coomassie staining intensity of the residual non-degraded IL-4 protein using the software ImageLab (Bio-Rad). The “proteolytic half-life” of each IL-4 protein was then calculated by one-phase decay using sPrism software (Table 3; see also Figure S11). Mutations F82D and R121C did not affect proteolytic sensitivity compared to wild-type IL-4 (see also Table S4). Non-glycosylated *E. coli*-derived proteins such as IL-4 F82D were highly susceptible to trypsin proteolysis and under these conditions exhibited rather short proteolytic half-lives of about 1 h. The HEK293 cell-derived IL-4 variant N38Q lacking all N-glycosylation was also degraded rather rapidly (*t*_1/2_ 2.4 h). Interleukin-4 F82D obtained from HEK293 cells, which carries a single complex N-glycan, exhibited a proteolytic half-life of about 4.5–5 h, indicating that a single glycan moiety already improved proteolytic stability (Table 3).

**Table 3:** Proteolytic Stability of Differently Glycosylated IL-4 Variants^a^

IL-4 variant	amount of N-glycans	half-life [h]
F82D (*E. coli*)	0	0.9 ± 0.01
F82D N38Q (HEK293)	0	2.4 ± 0.1
F82D (HEK293)	1	4.7 ± 1.7
F82D Q20N (HEK293)	2	10.5 ± 0.6
F82D Q20N T28N-K61N (HEK293)	4	25.4 ± 2.8
WT (*P. pastoris*)	1	2.6 ± 0.2
WT (HEK293)	1	4.9 ± 0.7
WT (*E. coli*)	0	1.7 ± 0.1

a Resistance to *in vitro* proteolysis was determined for IL-4 proteins with different level of glycosylation. Proteins were hydrolyzed with trypsin and degradation at different times was monitored using SDS-PAGE analysis. The level of the non-degraded IL-4 protein was determined by densiometric analysis of Coomassie-stained gels and fitted by an equation of exponential decay to yield values of proteolytic half-life. Mean and standard deviation from two independent experiments are shown.

Conformingly, variants harboring two (Q20N and the natural N-glycosylation site at Asn38) or four N-glycans (Q20N T28N-K61N and Asn38) showed strongly enhanced stability against degradation by trypsin (*t*_1/2_ 10.5 and 25 h, respectively). This clearly indicates that the proteolytic stability of IL-4 can be increased considerably *in vitro* by additional N-glycans using HEK293 cells as the expression system. Importantly, the size of the N-glycan moiety does not seem to be the determining factor of proteolytic stability, as IL-4 produced in the yeast *Pichia pastoris*, which carried a single large N-glycan of about 20 kDa size (determined by SDS-PAGE, see Figure S12), had a proteolytic half-life of only 2.6 h. Hence, albeit the yeast-derived IL-4 protein contained an N-glycan moiety about 8–10 times the size of the N-glycan moiety attached by mammalian cells (average N-glycan size is about 2–3 kDa), HEK293 cell-derived IL-4 carrying a single N-glycan exhibited similar proteolytic stability. This discrepancy might possibly be due to the difference in the glycan architecture and composition, as mammalian cells produce N-glycans of hybrid or complex type usually containing terminal negatively charged sialic acid residues, whereas yeast produces proteins attached to poly-mannose glycans not shielded by sialic acid end groups.^53^

## Discussion

In this study, we established different reaction schemes for chemical glycoengineering, which allowed site-specific conjugation of amine- or thiol-functionalized carbohydrate molecules to IL-4 harboring an unpaired cysteine. In this novel setup, a carbohydrate molecule was chemically introduced at an engineered site to block receptor binding ultimately conferring an inhibitory effect and converting the protein into an antagonist. Hence, these IL-4 F82D R121C conjugates were highly effective IL-4 antagonists *in vitro*, more potent than the benchmark Pitrakinra. By developing a procedure allowing chemical glycosylation during refolding of the (insolubly expressed) IL-4 protein, we obtained a fast strategy that neither requires enzymatic deglutathionylation nor thiol-activation for efficient coupling. Compared to other strategies, this scheme allows for time-efficient synthesis of chemically glycosylated IL-4 in high yield with almost 100% conjugation efficiency, thereby decreasing the number of purification steps. Furthermore, this strategy allows the coupling of the glycan moiety directly with the protein without the need for a (bifunctional) linker. As the latter has a certain minimal length due to the steric requirement of the chemical groups needed for conjugation a linker-mediated conjugation approach will inevitably display the glycan moiety in a hapten-like manner. This, however, might lead to recognition of this unusual chemical structure by the immune system and trigger unwanted immune responses against the therapeutic. With minor adjustments, the direct chemical glycosylation strategy could also be applied to HEK293 cell-derived (multi)glycosylated IL-4 F82D R121C variants containing up to four engineered N-glycosylation sites. This provides a combination of chemical and biosynthetic glycoengineering in which antagonist functionality is implemented through chemical glycosylation and additional N-glycosylation adds complex N-glycan moieties to positively modulate pharmacokinetic properties such as *in vivo* half-life and immunogenicity.

*In vitro* proteolysis assays revealed that in comparison to non-glycosylated proteins such multi-glycosylated IL-4 antagonists not only maintain their very high inhibition efficacy but also acquire a ≥30-fold increased stability against proteolytic degradation through trypsin. While IL-4 is not a physiological substrate of trypsin *in vivo*, similar biophysical and protein chemical parameters that confer resistance to trypsin degradation will also increase stability against proteases, *e.g.*, matrix metalloproteinases (MMPs), which might be physiologically more relevant for IL-4 degradation. These data suggest that such glycoengineered IL-4 inhibitors/antagonists likely exhibit improved pharmacokinetics properties *in vivo* compared to the original drug Pitrakinra and therefore present alternatives to classical IL-4 inhibitors such as the neutralizing anti-IL-4Rα antibody Dupilumab.

From a long-term perspective, the conjugation of complex human-like N-glycan structures to proteins purely carried out by chemical procedures will be the ultimate goal making the expression in eukaryotic expression hosts dispensable and allowing the production of chemically highly defined protein glycoconjugates. Obtaining a defined homogeneous conjugate would also allow the use of tailored glycan structures that can be used to modulate targeting of the pharmaceutical compound to desired target sites, facilitate the prediction, or modulate metabolic degradation, thereby enabling fine-tuning of pharmacokinetic properties. In a proof-of-principle experiment, the refolding strategy presented here readily allowed multi-site glycoconjugation of IL-4 cysteine variants. Given that an automated synthesis of complex glycans might be realized in the near future, this chemical glycoengineering will facilitate the preparation of pharmacologically optimized IL-4 antagonists but might also be applied for the production of other glycoproteins.

## Experimental Procedures

### Production of IL-4 in *E. coli* for Site-Directed Chemical Glycosylation

The cDNA encoding IL-4 variants were synthesized by two-step PCR mutagenesis (oligonucleotide sequences see Table S3). Expression, purification, and enzymatic deglutathionylation of *E. coli*-derived IL-4 cysteine variants were performed as described.^33^

### Coupling of IL-4 Cysteine Variants to Amine-Containing Carbohydrates via the Bifunctional Crosslinker SMCC

The crosslinker SMCC (succinimidyl-4-(*N*-maleimido-methyl)-cyclohexane-1-carboxylate) was dissolved at 100 mM in acetonitrile. A 400 mM glucosamine solution was prepared in 100 mM sodium phosphate and 4 mM EDTA, pH 8.0. In the first approach, these two solutions were mixed in volumetric 1:1 ratio, which resulted in a reaction mixture with a pH of 6.5. This mixture was then incubated at 21 °C for 2 h to allow for coupling of the amino group of glucosamine to the carboxylate group of SMCC. The SMCC-glucosamine conjugate was purified by reversed-phase HPLC (RP-HPLC) using a C18-column (μRPC C2/C18 ST 4.6/100, GE Healthcare) employing a gradient of 0.1% TFA/H_2_O to 100% acetonitrile. The coupling product was eluted at 55–60% acetonitrile. Conjugate-containing fractions were pooled and freeze-dried. In a revised setup glucosamine-HCl was dissolved in 200 mM sodium phosphate, 8 mM EDTA pH 8.0, and the pH of the glucosamine solution was adjusted to pH 7.2 by adding 1 M sodium hydroxide dropwise. This glucosamine-phosphate solution was then reacted with 100 mM SMCC solution in acetonitrile or alternatively with SMCC dissolved in dimethylsulfoxide. The alternative setups were reacted and purified as described above. Deglutathionylated IL-4 containing an unpaired cysteine was dissolved in 100 mM sodium phosphate (pH 6.5), glucosamine-SMCC conjugate was dissolved in acetonitrile at 5 mM concentration and both solutions were mixed in a 1:100 molar ratio (protein:gluco-SMCC conjugate). After incubating at 21 °C for 2 h, the IL-4 SMCC glycoconjugate was purified by RP-HPLC employing a C4 column (Jupiter C4, 10 μm, 250 mm × 10 mm, Phenomenex, Germany) and a gradient of 0.1% TFA in H_2_O to 100% acetonitrile in five column volumes.

### Coupling of IL-4 Cysteine Variants to Thiol-Carbohydrates Using Phenylselenyl Bromide Activation

Deglutathionylated IL-4 protein containing an unpaired cysteine was dissolved in 10 mM CHES, 70 mM MES, and 2 mM CaCl_2_ (pH 9.5) to a concentration of 66 μM. Phenylselenyl bromide was dissolved at 100 mM in acetonitrile. The reaction was started by mixing the phenylselenyl bromide and protein solutions in a 40:1 molar ratio. The mixture was incubated at 21 °C for 1 h. Non-reacted phenylselenyl bromide and by-products were removed by size exclusion using a PD10 column (GE Healthcare). Protein-containing fractions were pooled and stored at −20 °C. Thiol-glycans were dissolved in water at 30 mM concentration. A 66 μM protein–phenylselenyl conjugate in 10 mM CHES, 70 mM MES, and 2 mM CaCl_2_ (pH 9.5) was supplemented with 3 mM thiol-glycan and the mixture was then incubated for 20 min at 21 °C. Yield could be increased if additional thiol-carbohydrate (final concentration 6 mM) was added after 20 min and incubation at 21 °C was extended for another 20 min. The IL-4–carbohydrate conjugate was purified by RP-HPLC as described above.

### Direct Coupling of Thiol-Carbohydrates to IL-4 Cysteine Variants during Refolding

The IL-4 protein (*E. coli*-derived IL-4 inclusion bodies or from expression in HEK293 cells; for expression in HEK293 cells see the Supporting Information) was denatured using 5.3 M GuHCl, 100 mM Tris–HCl (pH 8.0), 1 mM DTT at a protein concentration of about 1–1.5 mg/mL (bacteria-derived insoluble protein) or 1 mg/mL (HEK293 cell-derived protein). After stirring for 2 h at 21 °C the mixture was centrifuged (35,000*g*, 4 °C, 30 min) and added to 25 volumes of refolding buffer (1 M arginine, 50 mM Tris–HCl, pH 8.0, 5 mM EDTA, 1 mM thiol-glycan). The refolding reaction was incubated at 4 °C for 72 h. Thereafter the refolding mixture of *E. coli*-derived IL-4 proteins was dialyzed twice for 12 h at 4 °C against 10 volumes of 25 mM ammonium acetate pH 5.0. Refolded *E. coli*-derived IL-4 glycoconjugates were purified by cation exchange chromatography (HiTrap CM Sepharose FF, GE Healthcare) using a gradient 0–1.5 M NaCl in 25 mM ammonium acetate (pH 5.0). Thereafter RP-HPLC was performed using a C4 column (Jupiter C4, 10 μm, 250 mm × 10 mm, Phenomenex, Germany) and a gradient of 0.1% TFA in H_2_O to 80% acetonitrile in three column volumes. The refolding mixture for HEK293 cell-derived IL-4 glycoconjugates was instead dialyzed twice for 12 h at 4 °C against 10 volumes of 50 mM sodium phosphate and 300 mM NaCl (pH 8.3). His-tagged HEK293 cell-derived IL-4 glycoconjugates were then isolated using immobilized metal ion affinity chromatography (IMAC) employing a 5 mL HisTrap Excel column (GE Healthcare) and a gradient of 10–500 mM imidazole in 50 mM sodium phosphate (pH 8.3) and 300 mM NaCl. Protein-containing fractions were pooled and dialyzed against 1 mM HCl.

### Cellular Assays for IL-4 Bioactivity

TF-1 cells (ATCC CRL2003) were maintained in RPMI-1640 (Gibco), 10% heat-inactivated fetal calf serum (FCS, Biochrome), 100 μg/mL streptomycin, 100 U/mL penicillin, and 8 ng/mL human GM-CSF at 37 °C and 5% CO_2_ in a humidified atmosphere. The day prior to the assay, cells were diluted 1:1 with the fresh medium. Cell densities were adjusted to (3–4) × 10^5^ cells/mL in the assay medium (RPMI-1640 without phenol red and without GM-CSF supplementation) and the cells were incubated for 4 h at 37 °C. To determine biological activities (EC50), serial dilutions of IL-4 proteins were performed in the assay medium in 96-well flat-bottom microtiter plates. Per well, 100 μL assay medium containing the respective IL-4 protein was added and plates were warmed to 37 °C. Then, 100 μL of cell suspension was added per well and plates were incubated for 72 h. The metabolic rate was quantified by adding 10 μL of resazurin solution (0.15 mg/mL in PBS) per well. In the presence of NADH or NADPH, resazurin is converted to resorufin, which is quantified by absorbance spectroscopy at 571 nm. After incubation for 4 h at 37 °C, absorbance was measured at 571 nm (background at 749 nm). For quantification, signal intensities were normalized to the control, which was 50 pM IL-4 WT. For competition experiments to determine the half-maximal inhibitory concentration (IC50), a serial dilution of the IL-4 antagonist protein was prepared in the assay medium containing 100 pM wild-type IL-4.

HEK-Blue IL-4/IL-13 cells (Invivogen) were maintained in DMEM glutamax (Gibco), 10% heat-inactivated FBS, 100 μg/mL streptomycin, 100 U/mL penicillin, 100 μg/mL zeocin, 10 μg/mL blasticidin at 37 °C, and 5% CO_2_ in a humidified atmosphere. To determine biological activities of IL-4 proteins serial dilutions were performed in the assay medium (growth medium without phenol red) in 96-well flat-bottom microtiter plates. Per well, 100 μL of the assay medium containing the IL-4 protein was added and plates were warmed to 37 °C. HEK-Blue cells were adjusted to 1 × 10^5^ cells/mL in the assay medium and 100 μL of the cell suspension was added to each well. After 20–24 h of incubation at 37 °C, the supernatant was collected and a p-nitrophenyl phosphate assay was performed allowing quantification of SEAP activity measuring absorbance at 405/630 nm. For competition experiments to determine the half-maximal inhibitory concentration (IC50) a serial dilution of the IL-4 antagonist protein was prepared in the assay medium containing 100 pM wild-type IL-4.

### Proteolytic Stability Assay

Proteins were dissolved in 50 mM Tris–HCl, 1 mM CaCl_2_, pH 7.6, and adjusted to a concentration of 30–32 μM. Five hundred microliters of protein solution were mixed with 10 μg of trypsin (sequencing grade trypsin, Promega, catalog number V5111) dissolved at 40 μM in resuspension buffer (Promega), and the proteolysis setup was incubated at 37 °C. At different time points, samples were taken, mixed with 1:20 (v/v) protease inhibitor cocktail set III (Calbiochem), and frozen at −20 °C. For each protein sample, 5 μg were subjected to SDS-PAGE analysis. After Coomassie staining gels were analyzed using ImageLab software (Bio-Rad) using the volume tool. Band intensities were normalized to protein bands of the molecular weight standard. Values were analyzed statistically using the two-tailed *t*-test for independent samples (the threshold for the declaration of statistical significance: *P* value < 0.05).

## Supporting Information Available

Chemicals, protocols for removal of the non-conjugated IL-4 protein with iodoacetyl-activated agarose, recombinant expression of IL-4 in HEK293 cells, mass spectrometry analysis, surface plasmon resonance (SPR) analysis, and periodic-acid–Schiff staining of glycosylated proteins. Data for RP-HPLC purification of SMCC glycoconjugate, mass spectrometry, SPR data, enzymatic hydrolysis of N-linked oligosaccharides in glycoengineered IL-4 variants, SDS-PAGE analysis, and trypsin proteolysis experiments (PDF)

## Abbreviations

IL: Interleukin
Th: T-helper cell
Ig: immunoglobulin
JAK: Janus kinase
STAT: Signal Transducers and Activators of Transcription
VCAM 1: Vascular Cell Adhesion Molecule 1
PEG: poly(ethylene glycol)
GSH: glutathione reduced
GSSG: glutathione oxidized
SMCC: succinimidyl-4-(*N*-maleimidomethyl)cyclohexane-1-carboxylate
NHS: *N*-hydroxysuccinimide ester
RT: room temperature
SPR: surface plasmon resonance
HPLC: high-pressure liquid chromatography
CHES: *N*-cyclohexyl-2-aminoethanesulfonic acid
MES: 2-(*N*-morpholino)ethanesulfonic acid
GuHCl: guanidine hydrochloride
DTT: dithiothreitol
EDTA: ethylenediaminetetraacetic acid
GM-CSF: granulocyte macrophage colony-stimulating factor
EC_50_: half-maximal effective concentration
IC_50_: half-maximal inhibitory concentration
SEAP: secreted alkaline phosphatase
