# Supplementary material for: Design of Glycoengineered IL-4 Antagonists Employing Chemical and Biosynthetic Glycosylation
Source: ACS Omega. 2023 Jul 5;8(28):24841–52. doi: 10.1021/acsomega.3c00726 (PMC10357448; doi:10.1021/acsomega.3c00726)
Supplement: Supplementary file 1 — ao3c00726_si_001.pdf [file ao3c00726_si_001.pdf]

Supporting information

Design of glycoengineered IL-4 antagonists employing chemical and biosynthetic glycosylation

Sarah Thomas[†], Juliane E. Fiebig[†], Eva-Maria Kuhn[†], Dominik S. Mayer[†], Sebastian Filbeck[†], Werner Schmitz[‡], Markus Krischke[§], Roswitha Gropp[⊥] and Thomas D. Mueller[†,*]

† Department of Molecular Plant Physiology and Biophysics, Julius-von-Sachs Institute of the University Wuerzburg, Julius-von-Sachs Platz 2, D-97082 Wuerzburg, GERMANY

‡Department of Biochemistry and Molecular Biology, Biocenter of the University Wuerzburg, Am Hubland, D-97074 Wuerzburg, GERMANY

§ Department of Pharmaceutical Biology, Julius-von-Sachs Institute of the University Wuerzburg, Julius-von-Sachs Platz 2, D-97082 Wuerzburg, GERMANY

⊥Department of General- Visceral-, Vascular- and Transplantation Surgery, Hospital of the LMU, Nussbaumstr. 20, 80336 Munich, Germany

* to whom correspondence should be addressed: mueller@biozentrum.uni-wuerzburg.de

Contents

General information S3

Chemicals S3

Removal of non-conjugated IL-4 protein using iodoacetyl-activated agarose S3

Recombinant expression of IL-4 in HEK293 cells S4

Electrospray Ionization Mass Spectrometry Analysis (ESI-MS) S5

Surface Plasmon Resonance (SPR) Interaction Analysis S5

Periodic acid-Schiff (PAS) staining of glycosylated proteins S7

Table S1: Oligonucleotides used to introduce cysteine residues and additional N-glycosylation sites into the IL-4 coding sequence S8

Table S2: Carbohydrate compounds used to generate glycoconjugates from IL-4 cysteine variants S9

Table S3: Theoretical and experimental molecular weights of IL-4 cysteine analogues S10

Table S4: Proteolytic stability of IL-4 variants designed for glycoengineering S11

Figure S1: Transient expression of IL-4 F82D N38Q R121N K123S in HEK293 cells. S12

Figure S2: Determination of incubation time for thiol group release by glutaredoxin. S13

Figure S3: RP-HPLC analytics of SMCC-glucosamine conjugation S15

Figure S4: RP-HPLC purification of IL-4 F82D R121C conjugated to SMCC-glucosamine S16

Figure S5: Mass spectrometry analysis of IL-4 F82D R121C glycoconjugates S17

Figure S6: SDS-PAGE analysis of IL-4 F82D R121C glycoconjugates S18

Figure S7: In vitro interaction analysis using surface plasmon resonance (SPR) S19

Figure S8: Quantitative Real Time PCR of IL-4 receptor transcript levels in TF1 cells S20

Figure S9: Enzymatic hydrolysis of N-linked oligosaccharides in glycoengineered IL-4 variants S21

Figure S10: Dose-response curves of glycoengineered IL-4 variant F82D Q20N T28N K61N R121C-Glc in TF1 cells S23

Figure S11: Analysis of time-dependent degradation of IL-4 proteins and glycoconjugates by trypsin S23

Figure S12: SDS-PAGE analysis of wildtype IL-4 expression from E. coli, yeast and HEK293 cells S24

Additional references S25

General Information

Chemicals

E. coli glutaredoxin (GRX-01) was obtained from Cayman Chemical, glutathione and NADPH were purchased from Carl Roth GmbH (Karlsruhe, Germany), yeast glutathione reductase (GR) was obtained from Sigma-Aldrich (St. Louis, MO, USA). Succinimidyl 4-(N-maleimidomethyl) cyclohexane-1-carboxylate (SMCC) was purchased from Apollo Scientific (Cheshire, UK). Protease Inhibitor Cocktail Set III and D(+)glucosamine hydrochloride were obtained from Merck (Darmstadt, Germany). Phenylselenyl bromide, 1-thio-β-D-glucose sodium salt and 1-thio-β-D-glucose tetraacetate were ordered from Sigma-Aldrich (St. Louis, USA). Sequencing grade trypsin was acquired from Promega (Fitchburg, USA).

Removal of non-conjugated IL-4 protein using iodoacetyl-activated agarose

Iodoacetyl-activated agarose beads (SulfoLink™, Thermo Fisher Scientific) were used to remove non-reacted IL-4 protein harboring free sulfhydryl-groups derived from chemical glycosylation. One mg of IL-4 glycoconjugate mixture was dissolved in 50 mM Tris-HCl, 1 mM EDTA pH 8.0 and subjected in batch mode to 1 mL of activated resin. The suspension was repeatedly mixed and incubated at 21°C for 45 min to ensure coupling of any IL-4 protein containing a free thiol group to the resin. The suspension was then filled into an empty column jacket and the flow through was collected. Thereafter, the resin was washed three times with one column volume coupling buffer and the flow through was again collected to completely elute IL-4 glyco-conjugates containing no free thiol group from the resin. Fractions containing IL-4 glycoconjugate were pooled, dialyzed against 1 mM HCl and the protein was freeze-dried for storage.

Recombinant expression of IL-4 in HEK293 cells

For production of IL-4 and variants carrying biosynthetic complex N-glycans the HEK293 cell lines Freestyle293 (Thermo Fisher Scientific) and Expi293 (Thermo Fisher Scientific) were used, which were maintained in suspension in the respective FreeStyle293 or Expi293 medium following the manufacturer`s recommendation. Additional N-glycosylation sites were introduced into the IL-4 coding sequence using two-step PCR mutagenesis (oligonucleotide sequences see Table S1). The modified IL-4 cDNA were then cloned into a modified version of the expression vector pHLsec[1]. The 5' end of the IL-4 gene (encoding for residues His25 to Ser153) was fused to a DNA fragment encoding a hexahistidine sequence followed by a short linker (SSG) and an 8mer sequence (LVPRGSTG) harboring a recognition site for the endopeptidase thrombin. This construct allowed secretion of the IL-4 protein into the medium, from where it could be isolated by immobilized metal ion affinity chromatography via its N-terminal His-tag, which could be removed by proteolysis with thrombin. Plasmid DNA were transiently transfected into HEK293 cells using polyethyleneimine (PEI)[2]. For transfection of FreeStyle293 or Expi293 cells two solutions were made: for each mL cell culture (cell density of FreeStyle293 cells: 0.75×10^6 cells/mL; cell density of Expi293 cells: 2.5×10^6 cells/mL) 35 µL Opti-MEM I medium (Gibco) containing 1 µg DNA (solution A) were prepared as well as solution B comprising 35 µl Opti-MEM I containing 2 µg PEI (25 kDa linear, Poly-science) (also per mL cell culture). Both solutions were mixed, incubated for 25 min at 21°C and added slowly to the cells. After 5 days, the cell culture medium usually comprising 800 mL (FreeStyle293 cells) or 400 mL (Expi293 cells) was clarified by centrifugation (800 xg, 4°C, 25 min), the supernatant then was dialyzed against 50 mM sodium phosphate, 300 mM NaCl pH 8.3 (running buffer), and submitted to immobilized metal ion affinity chromatography employing a 5 mL HisTrap™ excel column (GE Healthcare). IL-4 proteins were then eluted in a step gradient with the buffer supplemented with 500 mM imidazole. Protein-containing fractions were identified by SDS-PAGE analysis, pooled and dialyzed against 1 mM HCl. The dialyzed protein was freeze-dried and stored at -80°C.

Electrospray Ionization Mass Spectrometry Analysis (ESI-MS)

ESI-MS analyses of protein samples were performed on a Q Exactive™ Hybrid Quadrupole-Orbitrap™ mass spectrometer (Thermo Fisher Scientific). Freeze-dried protein samples were dissolved in methanol/water/acetic acid (49.5/49.5/1) and adjusted to a concentration of 2 µM. Direct sample injection was performed and ions were detected in positive mode from 500 to 2500 m/z. For evaluation, the FID signal (free induction decay) from 58 scans was deconvoluted to the single protonated ion mode using the software mMass (http://www.mmass.org).

ESI-MS analysis of low-molecular weight compounds, i.e. SMCC and SMCC-glucosamine conjugates, was done using ultra-performance liquid chromatography-tandem mass spectrometry (UPLC-MS-MS). For chromatographic separation a C18 column (Acquity UPLC BEH C18, 1.7 µm, 2.1 × 50 mm equipped with a 5 x 2.1 mm guard column; Waters; Milford, MA, USA) was employed using 0.1% formic acid and acetonitrile as solvents for a binary gradient. Elution was performed using a linear gradient from 3% to 10% acetonitrile at a flow rate of 0.25 mL/min in 7 min and a column temperature of 40°C. For ion detection, a full scan experiment was performed from 50 to 1500 m/z in the positive electrospray mode on a Waters Micromass Quattro Premier™ triple quadrupole mass spectrometer. Data was processed using MassLynx Software.

Surface Plasmon Resonance (SPR) Interaction Analysis.

All biosensor experiments were carried out employing a ProteOn^{TM} XPR 36 system (BioRad) using 10 mM HEPES, 300 mM NaCl, 3.4 mM EDTA, 0.005% (v/v) Tween-20, pH 7.4 (HBST300) as running buffer. Experiments were performed at 25°C using the single kinetics setup specific for the ProteOn^{TM} XPR36 system, which allows acquisition of ligand-analyte interactions with six different analyte concentrations simultaneously. For biosensor preparation, the extracellular domain of human IL-4Rα (IL-4RαECD) comprising residues Lys27 to Leu233 was expressed in Baculovirus-transfected HighFive insect cells. The expression construct was equipped with an N-terminal gp64 signal peptide for secretion into the medium followed by a 13 amino acid long ybbR peptide

sequence (MVDSLEFIASKLA) and a short 3mer GSF linker ahead of the IL-4Rα ectodomain sequence, which allows site-specific enzymatic conjugation of a biotin to the first serine residue within this sequence. For purification from cell culture supernatant ybbR-IL-4RαECD was equipped with a deka-histidine sequence at the C-terminus and an eight amino acid sequence N-terminal of the polyhistidine sequence harboring a recognition site for the endopeptidase PreScission to allow proteolytic removal of the dekahistidine sequence. Expression and purification of ybbr-IL-4RαECD followed the published protocol [3]. For site-specific biotinylation ybbR-IL-4Rα ECD protein (concentration 10 μM) dissolved in 50 mM HEPES pH 7.4, 10 mM $MgCl_2$ was mixed with CoA-biotin (New England Biolabs) in a 1:1 molar ratio and the mixture was incubated in the presence of 0.5 μM Sfp phosphopantetheinyl transferase (New England Biolabs) first at 22°C for 2 h and then at 8°C overnight. Non-reacted CoA-biotin was removed by repetitive ultrafiltration (Vivaspin 4, 5K MWCO PES, Sartorius) using PBS as buffer. Biotinylation was confirmed by Western blot analysis using horseradish-coupled streptavidin for detection. For sensor chip preparation, a ProteOn™ GLC biosensor (BioRad) was activated using sulfo-NHS (N-hydroxysulfosuccinimide) and EDAC (1-ethyl-3-(3-dimethylaminopropyl)-carbodiimide) following manufacturer's (BioRad) recommendation. Thereafter activated chip was perfused with 40 μg/mL streptavidin dissolved in 10 mM sodium acetate pH 4.5 until an immobilization density of about 3000 RU was obtained. This streptavidin-coated sensor chip was then perfused with site-specifically biotinylated IL-4RαECD protein until IL-4RαECD was immobilized at a density of 200-300 RU. For interaction analysis IL-4 proteins (and glycan-conjugates thereof) were injected onto this IL-4Rα ECD biosensor employing six different concentrations (5.0 nM, 2.5 nM, 1.25 nM, 0.625 nM, 0.313 nM and 0.157 nM in HBST300) and a flowrate of 100 μL/min. Non-specific binding of the analytes to the sensor matrix and bulk face effects were removed from interaction data by subtracting the SPR signal derived from the interaction of the analyte with a control flow channel coated only with streptavidin. Binding kinetics were determined by regression analysis of the association and dissociation phase of the sensogram employing a simple 1:1 Langmuir-type interaction model.

Equilibrium binding constants K_D were calculated from the equation $K_D = k_{off}/k_{on}$. Regression analysis was performed with all fitting parameters treated global, binding parameters were only used for interpretation if the chi^2 for fitting analysis was less than 10% of the signal amplitude. All SPR measurements were performed as independent triplicate.

Periodic acid-Schiff (PAS) staining of glycosylated proteins.

Periodic acid converts vicinal hydroxyl groups of sugars to aldehydes[4]. Subsequent treatment with Schiff's reagent (mixture of pararosaniline and sodium bisulfite) results in reaction of the glycan-aldehyde groups with the amino groups of the Schiff reagent thereby resulting in a magenta coloration. To stain an SDS-gel is first incubated in 50% (v/v) ethanol for 30 min at room temperature, the gel is then washed with water five times over a period of 10 min to remove all ethanol. The glycoproteins are then oxidized by incubating the gel for 30 min with 1 % (w/v) periodic acid in 3% acetic acid. Excess of the oxidizing reagent is removed by extensive washing with water. Then the gel is incubated twice for 10 min in 0.1% (w/v) sodium metabisulfite in 10 mM HCl and subsequently the gel is soaked for 1 h I Schiff's reagent (Carl Roth) in the dark. Then the gel is treated with 0.5 % (w/v) sodium metabisulfite in 10 mM HCl for at least 4 times over a period of at least 2 h to ensure sufficient coloration of the gel. For fixation and drying the gel is then soaked in 30% (v/v) methanol, 6 % (v/v) glycerol and dried between cellophane film.

Table S1: Carbohydrate compounds used to generate glycoconjugates from IL-4 cysteine variants.

compound	structure	abbreviation
SMCC-Glucosamine	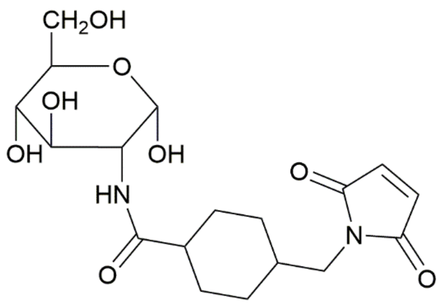	F82D R121C-SMCC-GlcN
1-thio-β-D-glucose sodium salt	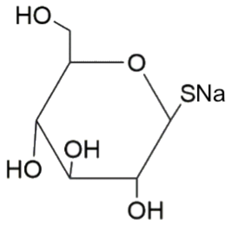	F82D R121C-Glc
1-thio-β-D-glucose tetraacetate	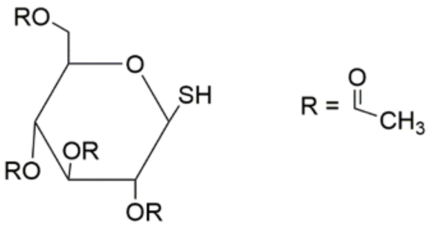	F82D R121C-4acGlc

Table S2: Theoretical and experimental molecular weights of IL-4 cysteine analogues

IL-4 variant	theoretical	observed	difference
F82D R121C-SH	15003.07	15003.59	0.52
F82D R121C-SMCC-GlcN	15401.43	15401.78	0.35
F82D R121C-Glc	15197.28	15197.61	0.33
F82D R121C-4acGlc	15365.44	15365.65	0.21
F82D N38C-Glc R121C-Glc	15380.53	15380.63	0.1
F82D N38C-4acGlc R121C-4acGlc	15716.84	15717.69	0.85
F82D R121C-Glc (HEK293 cell)	16463.51	16464.25	0.75

Theoretical and experimental molecular weight of the glycoengineered IL-4 F82D R121C analogues conjugated to different saccharide moieties as derived by the mass spectrometry analysis (see also Figure S2). For comparison, enzymatically deglutathionylated IL-4 F82D R121C-SH containing a free thiol group is included. Mass spectrometry analysis of the IL-4 variant F82D R121C protein derived from HEK293 cell expression and hence carrying a complex N-glycan at Asn38 was performed after hydrolysis of the N-glycan moiety with endoglycosidase PNGaseF. Abbreviations are SMCC-GlcN for SMCC-glucosamine conjugate, Glc for glucose and 4acGlc for glucose tetraacetate.

Table S3: Oligonucleotides used to introduce cysteine residues and additional N-glycosylation sites into the IL-4 coding sequence.

Name	Sequence
R121D Y124D_s	GACGATCATGGACGAGAAAGATTC
R121D Y124D_as	GAATCTTTCTCGTCCATGATCGTC
F82D_s	CAGCTGATCCGAGACCTGAAACGG
F82D_as	CGTTTCAGGTCTCGGATCAGCTG
**Introduction of cysteines**	
N38C_s	CTGCCTCCAAGTGCACAACTGAGAAG
N38C_as	CTTCTCAGTTGTGCACTTGGAGGCAG
R121C_s	GGCTAAAGACGATCATGTGTGAGAAATATTCAAAGTG
R121C_as	CACTTTGAATATTTCTCACACATGATCGTCTTTAGCC
**Introduction of N-glycosylation sites**	
Q20N_s	GCCTCACAGAGAACAAGACTCTGTGCACCG
Q20N_as	CGGTGCACAGAGTCTTGTTCTCTGTGAGGC
T28N_s	GCACCGAGTTGAACGTAACAGAC
T28N_as	GTCTGTTACGTTCAACTCGGTGC
K61N_s	CCACCATGAGAACGACACTCGCTGC
K61N_as	GCAGCGAGTGTCGTTCTCATGGTGG
R121N_K123S_s	CTAAAGACGATCATGAACGAGAGCTATTCAAAGTGTTC
R121N_K123S_as	GAACACTTTGAATAGCTCTCGTTCATGATCGTCTTAG
N38Q_s	CTGCCTCCAAGCAGACAACTGAGAAG
N38Q_as	CTTCTCAGTTGTCTGCTTGGAGGCAG

Table S4: Proteolytic stability of IL-4 variants designed for glycoengineering.

IL-4 variant	Amount of N-glycans	Normalized half-life fold
WT (*E. coli*)	0	1 (1.5 ± 0.3 h)
F82D N38C (*E. coli*)	0	1.9 ± 0.5
F82D R121C (*E. coli*)	0	2.4 ± 0.2
F82D N38C R121C (*E. coli*)	0	1.5 ± 0.2
F82D Q20N-T28N-K61N (HEK293)	4	47.8 ± 9.8
F82D (HEK293)	1	8.6 ± 1.5

In order to show that the introduction of (an) additional cysteine residue(s) for chemical glyco-conjugation does not alter the proteolytic stability of IL-4 an additional set of proteolysis experiments similar as described in materials and methods were conducted. As a different supplier/lot of trypsin was used wildtype IL-4 (derived from *E. coli*), IL-4 F82D (Q20N T28N K61N) (harboring four N-glycosylation sites) and IL4 F82D (derived from HEK293 cells thus harboring one N-glycosylation site) were used as internal references to allow comparison of the proteolytic half-lifes determined for these set of proteins with those in Table 3. Briefly, 300 µl IL-4 protein solution with a concentration of 25 µM was mixed with six µl 40 µM trypsin sequencing grade (Roche, catalog number 11418475001) resulting in molar protein:protease ratio of about 30:1. The proteolysis setup was incubated at 37°C and samples of 30 µl were taken at 0 (starting point), 0.5, 1, 2, 4, 6, 8 24h (for glycosylated IL-4 proteins additional samples were taken at 30, 48 and 96h). The samples were immediately mixed with 1.5 µl protease inhibitor (Calbiochem Protease-Inhibitor-Cocktail-Set III. EDTA-free) and stored at -20°C. For analysis, 6 µl 6x SDS loading buffer were added, the samples were heated to 95°C for 5 min and 12 µl (= 4.5 µg) were subjected to SDS-PAGE. After Coomassie staining the gels were scanned using a flatbed scanner (Mikrotec) and the scans were analyzed using the Analyze Gels tool in the software ImageJ to quantify the protein bands on the SDS gels. The volume data from the protein bands derived were then fitted with a one

phase decay routine using Prism version 9 to yield half times for proteolytic degradation. Two independent proteolysis experiments were performed. The half-life of wildtype IL-4 reived from E. coli was set to 1 and the other half-life values were normalized to this value.

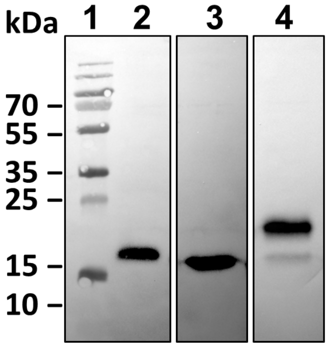

Figure S1: Transient expression of IL-4 F82D N38Q R121N K123S in HEK293 cells. Cell supernatants from transient expression of IL-4 protein in HEK293 cells were submitted to SDS−PAGE and Western blot analysis. IL-4 variants carrying an N-terminal hexahistidine-tag were detected using an anti-His antibody conjugated to horseradish peroxidase. 1: size standard; 2: cell culture supernatant of transient expression of IL-4 F82D N38Q; 3: cell culture supernatant of transient expression of IL-4 F82D N38Q R121N K123S; 4: cell culture supernatant of transient expression of IL-4 WT.

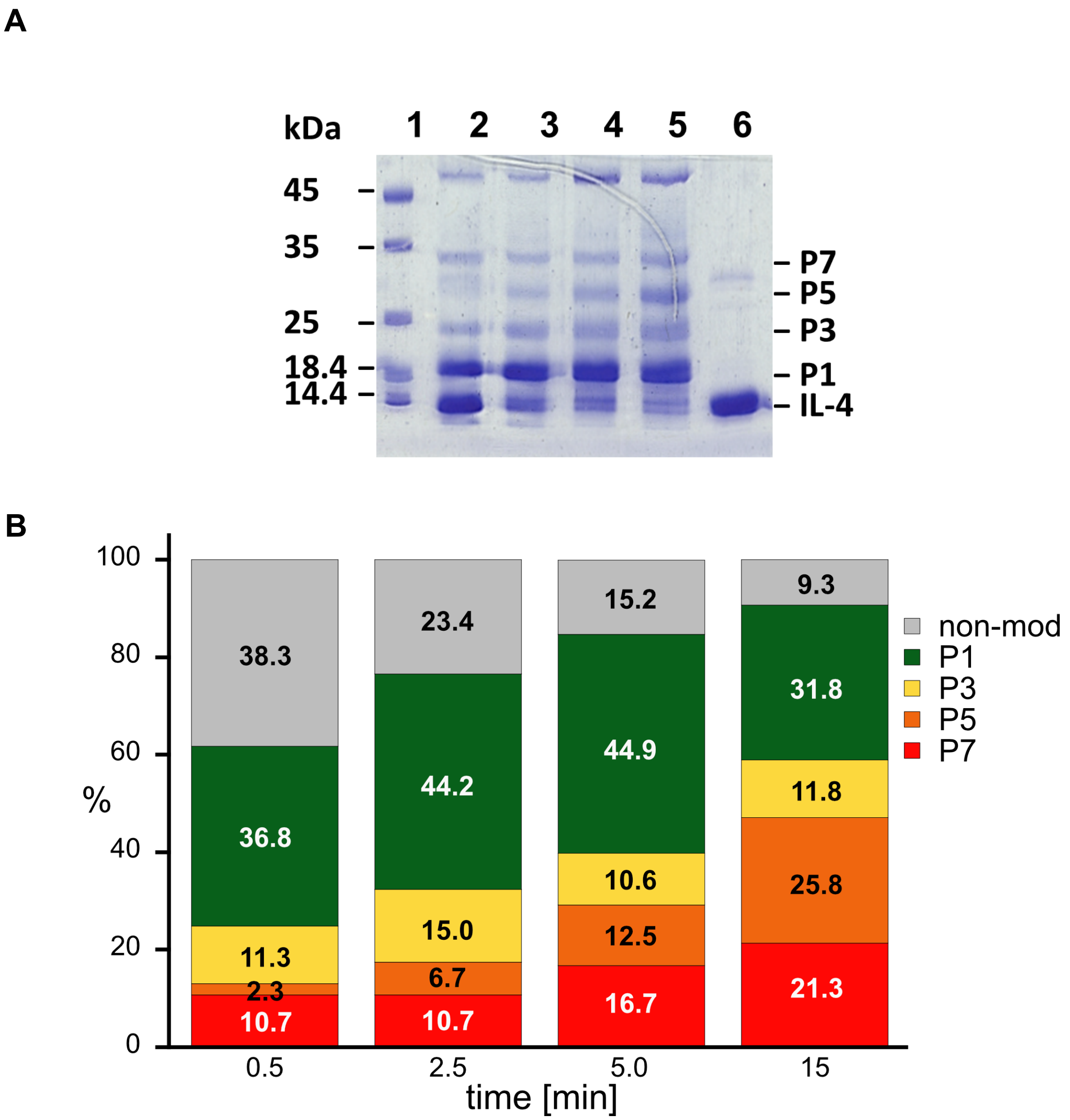

Figure S2: Determination of incubation time for thiol group release by glutaredoxin. (A) SDS-PAGE analysis of glutathionylated IL-4 variant F82D R121C after reaction with glutaredoxin-1. Samples were taken at different time points and incubated with 5 mM maleimide-PEG. 1: size standard; 2: after 0.5 min incubation with glutaredoxin-1; 3: after 2.5 min; 4: after 5 min; 5: after 15 min; 6: untreated control (not incubated with glutaredoxin-1). Electrophoretic mobility of mono-pegylated species is indicated with P1, multi-pegylated species are marked with P3, P5, and P7. (B) Quantitative analysis of glutaredoxin-mediated thiol group release. The SDS gel (A) was processed with ImageJ version 1.53 (http://imagej.nih.gov/ij), first the background of the gel was subtracted, then the lanes 2 to 5 of the gel were selected and gel band volumes were

determined with the analysis tool Analyse Gels and the Wand tool. The percentage of the individual non-, mono- and multi-pegylated species were calculated from the ratio of the volume of the respective gel band to the sum of all gel band volumes (shown in bold letters). The stacked bar plot shows that the non-pegylated IL-4 species decreases with incubation time, while mono- and multi-pegylated IL-4 increases. Upon 2.5 min incubation with glutaredoxin, the amount of mono-pegylated IL-4 reaches a plateau of about 44%, longer incubations times do not significantly increase the mono-pegylated IL-4 species but instead lead to an increase in the multi-pegylated IL-4 species P3, P5 and P7 as well as a high molecular weight pegylated species seen at the top of the SDS-gel.

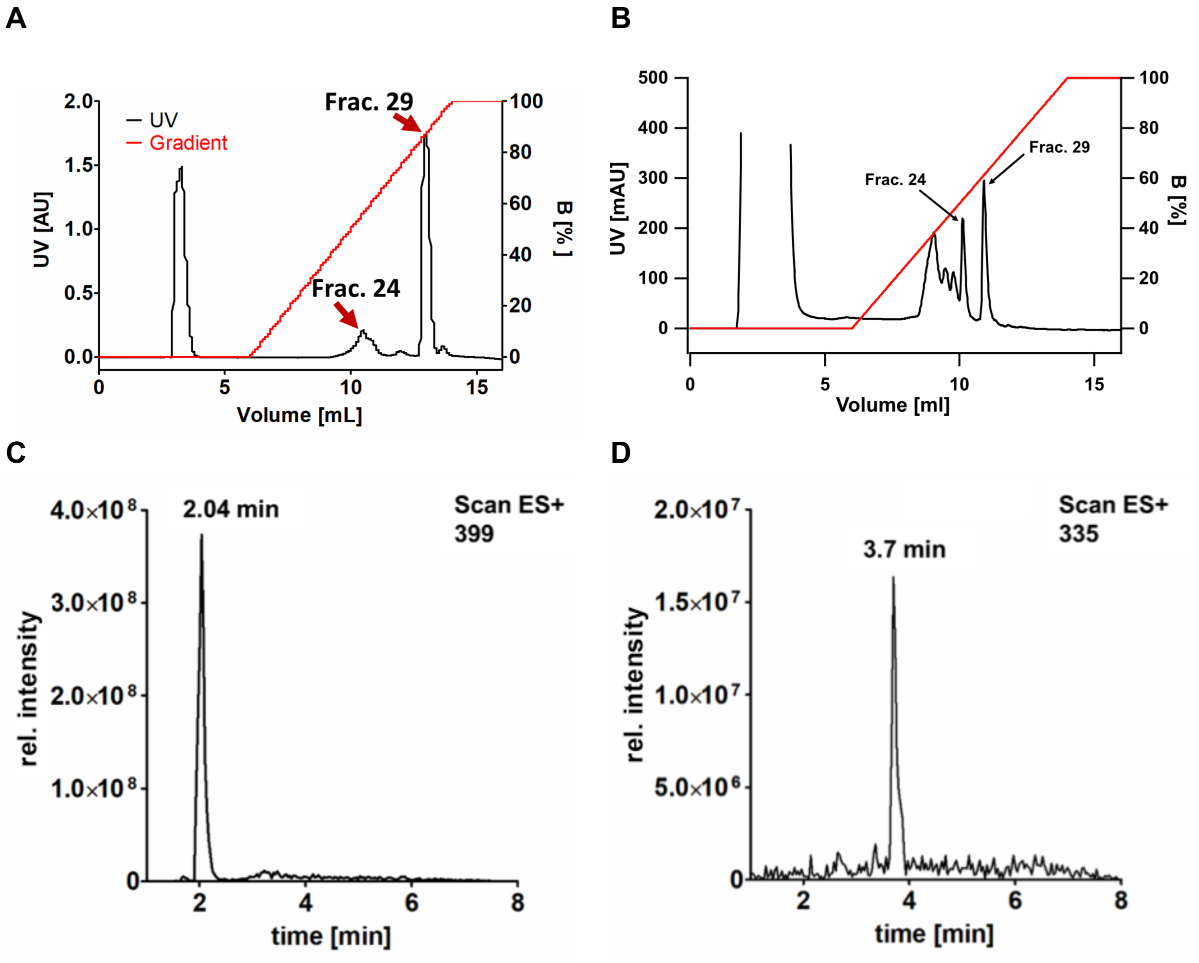

Figure S3: RP-HPLC analytics of SMCC-glucosamine conjugation. A) RP-HPLC chromatogram of the purification of glucosamine-SMCC from the initial reaction setup. The UV absorbance at 280 nm is shown as black line, the gradient of 0.1% trifluoroacetic acid in water to acetonitrile is indicated in red. Fraction 24 and 29 are indicated by arrows. B) RP-HPLC chromatogram of the purification of glucosamine-SMCC from an optimized reaction setup at pH 7.5. C) UPLC-MS analysis of RP-HPLC elution fraction 24 from purification shown in (A): the extracted ion chromatogram of an m/z value of 399, which corresponds to the theoretical mass of the SMCC-glucosamine conjugate, yielded one peak at a retention time of 2.04 min D) UPLC-MS analysis of RP-HPLC elution fraction 29 from purification shown in (A): the extracted ion chromatogram of an m/z value of 335, which is consistent with the theoretical mass of non-conjugated SMCC, yielded one peak at a retention time of 3.7 min.

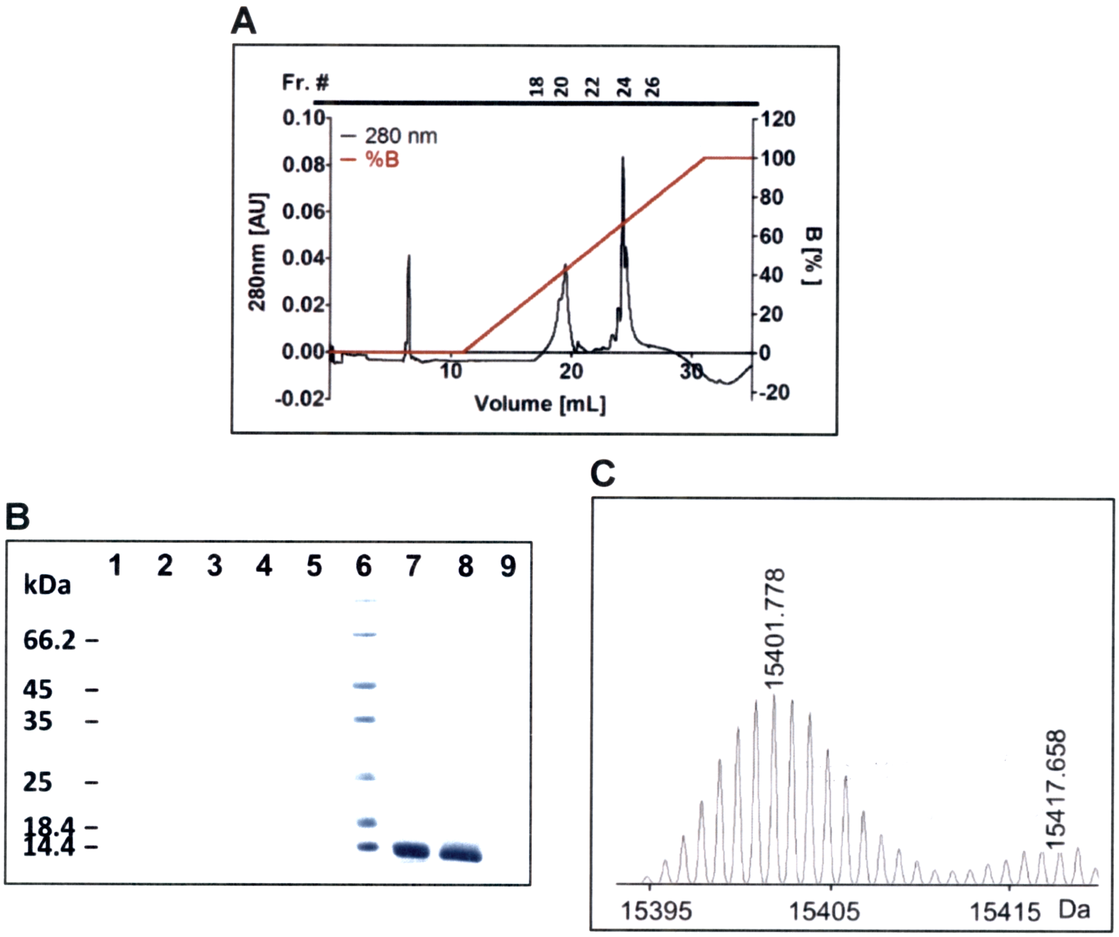

Figure S4: RP-HPLC purification of IL-4 F82D R121C conjugated to SMCC-glucosamine. A) RP-HPLC chromatogram of the purification of glucosame-SMCC conjugated IL-4 F82D R121C. B) SDS-PAGE analysis of the elution fractions, lanes 1-5: fractions 20 to 24; lane 6: size standard; lane 7: fraction 25; lane 8: fraction 26; lane 9: fraction 27. C) ESI-MS analysis of the purified IL-4 F82D R121C SMCC GlcN conjugate. Only protein consistent with the mass completely conjugated protein (15401 Da) containing one SMCC-glucosamine conjugated to the thioether linkage could be detected. Minor amounts of a species (+16 Da) representing a mono-oxygenated protein (15417 Da) are observed.

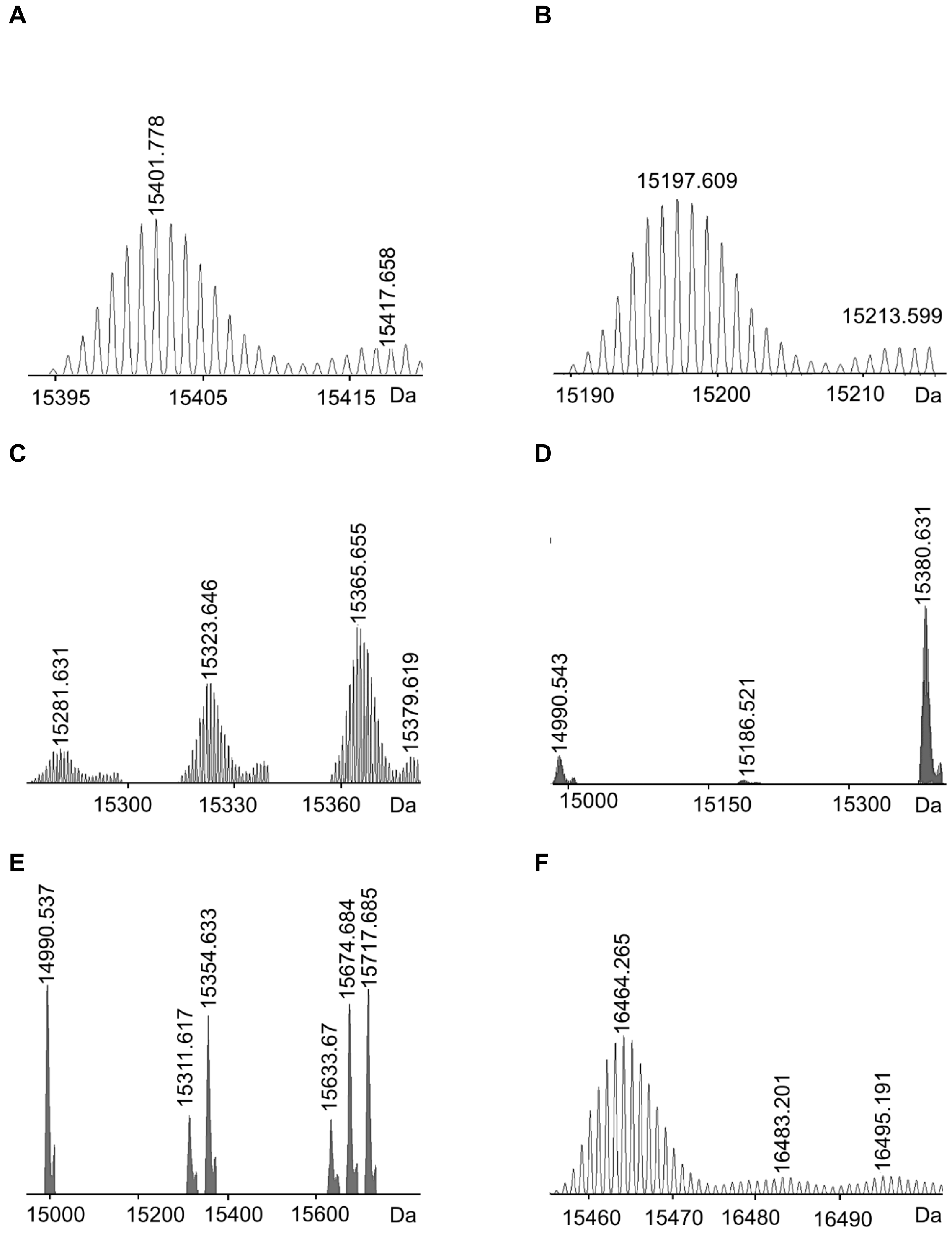

Figure S5: Mass spectrometry analysis of IL-4 F82D R121C glyco-conjugates. Minor species observed with a mass difference of +16 Da (e.g. see panels A and B) compared to the major signal represent mono-oxygenated species. Loss of acetyl groups (-42 Da) can be observed for IL-4

analogues conjugated to glucose tetraacetate (see panel C). A: IL-4 F82D R121C-SMCC-GlcN; B: IL-4 F82D R121C-Glc; C: IL-4 F82D R121C-4acGlc; D: IL-4 F82D N38C-Glc R121C-Glc (14990 Da: non-conjugated form; 15186 Da: single conjugated species containing one glucose molecule and one additional hydrogen atom at the remaining free thiol group); E: IL-4 F82D N38C-4acGlc R121C-4acGlc (14990 Da: non-conjugated with one additional disulfide bond; 15355 Da: single-conjugated species containing one glucose tetraacetate molecule and one additional hydrogen atom at the remaining free thiol group); F: IL-4 F82D R121C-Glc (HEK293 cell derived with the complex N-glycan at position 38 removed by hydrolysis with PNGase F).

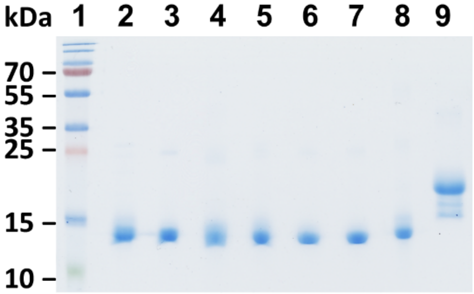

Figure S6: SDS-PAGE analysis of IL-4 F82D R121C glycoconjugates. SDS-PAGE analysis was performed under non-reducing conditions. 1: Molecular weight standard; 2: Wildtype IL-4; 3: IL-4 F82D N38C-Glc; 4: IL-4 R121D Y124D; 5: IL-4 F82D R121C-SH; 6: IL-4 F82D R121C-Glc; 7: IL-4 F82D R121C-4acGlc; 8: IL-4 F82D R121C-SMCC-GlcN; 9: IL-4 F82D R121C-Glc (HEK293 derived).

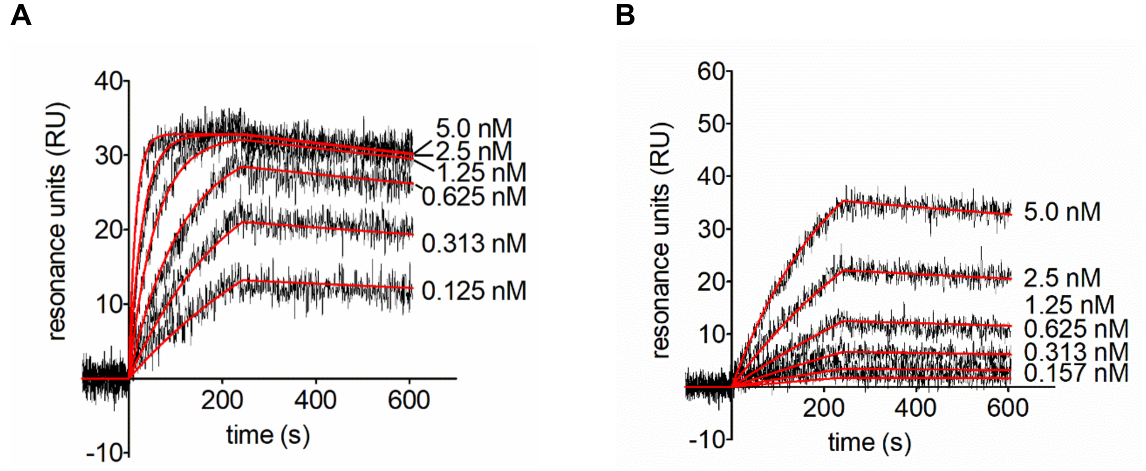

Figure S7: In vitro interaction analysis using surface plasmon resonance (SPR). For measurement of *in vitro* binding parameters using SPR, IL-4 glycoconjugates were perfused as analytes in six different concentrations over a ProteOn XPR36 GLC biosensor, which was coated with IL-4RαECD. Association of the analyte was monitored for 240 s (analyte injection starts at time point 0), then perfusion was stopped (time point 240 s) and running buffer was injected to monitor dissociation of the analyte from IL-4RαECD. Dissociation of IL-4 glycoconjugates was monitored for 300 s. Typical sensograms are shown of A) IL-4 F82D R121C-Glc (*E. coli* derived) and B) IL-4 F82D Q20N T28N K61N R121C-Glc (HEK293 derived).

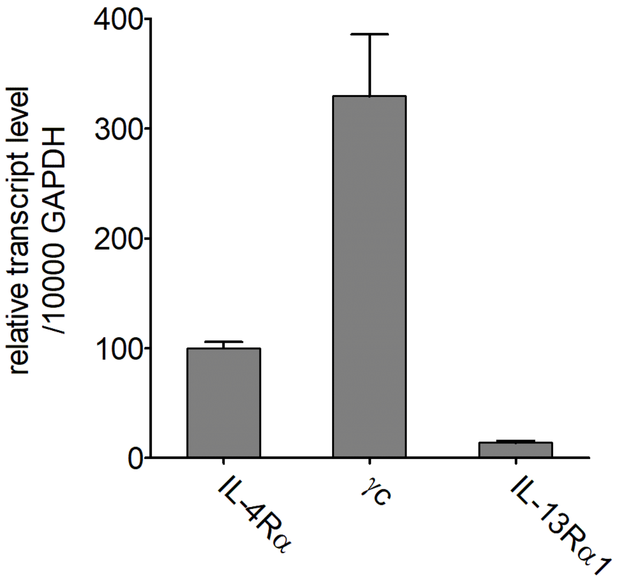

Figure S8: Quantitative Real Time PCR of IL-4 receptor transcript levels in TF-1 cells. Total mRNA was extracted from $2x10^7$ TF-1 cells using the Perfect Pure RNA Cultured Cell Kit (5Prime) and transcribed into cDNA using the RevertAid First Strand cDNA Synthesis Kit (Thermo Fisher Scientific). QPCR primers for IL-4Rα, IL-13Rα1, γc and GAPDH were purchased from Sigma Aldrich and qPCR reaction was performed using a Lightcycler™ (Roche, Switzerland) and the ABsolute QPCR SYBR Green Capillary Mix (Thermo Scientific, USA). For quantification, receptor-gene expression levels were normalized to 10 000 molecules GAPDH.

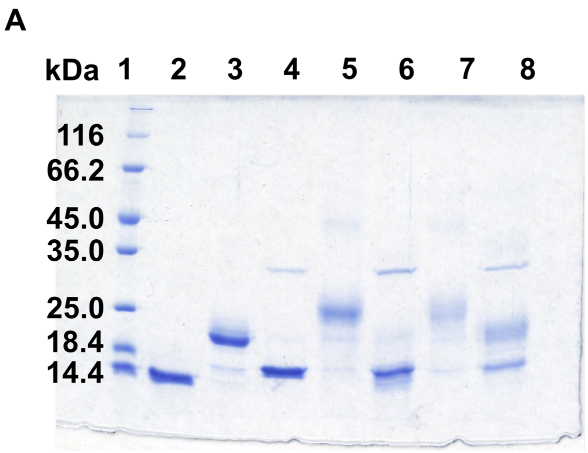

Figure S9: Enzymatic hydrolysis of N-linked oligosaccharides in glycoengineered IL-4 variants. To confirm that the N-glycosylation degrons (N-X-S/T motifs) that were introduced into IL-4 F82D variants are processed correctly and carry an N-linked oligosaccharide, IL-4 variants were expressed in the eucaryotic Expi293 (HEK293) cell expression system, purified and subjected to enzymatic removal using the endoglycosidase PNGaseF. Ten µg protein in 35.5 µl water were mixed with 4.5 µl master mix (0.5 µl PNGase F mixed with 4 µl PNGase F 10x buffer, Gibco PNGase F Glycan Cleavage Kit), mixed and incubated at 50 °C for 6 h. The cleavage was stopped by adding 6x SDS-loading buffer, the samples were heated to 95 °C for 5 min and subjected to SDS-PAGE. The SDS-gel was stained with Coomassie blue and dried between cellophane films. SDS-gel 1 (A): lane 1: molecular weight standard; lane 2: wildtype IL-4 from bacterial expression; lane 3: wildtype IL-4 from expression in Expi293 cells, untreated; lane 4: wildtype IL-4 from expression in Expi293 cells, treated with PNGase F; lane 5: IL-4 F82D Q20N from expression in Expi293 cells, untreated; lane 6: IL-4 F82D Q20N from expression in Expi293 cells, treated with PNGase F; lane 7: IL-4 F82D T28N from expression in Expi293 cells, untreated; lane 8: IL-4 F82D T28N from expression in Expi293 cells, treated with PNGase F. SDS-gel 2 (B): lane 1: molecular weight standard; lane 2: IL-4 F82D K61N from expression in Expi293 cells, untreated; lane 3: IL-4 F82D K61N from expression in Expi293 cells, treated with PNGase F. lane 4: IL-4 F82D (Q20N T28N K61N) from expression in Expi293 cells, untreated; lane 5: IL-4 F82D (Q20N T28N K61N) from expression in Expi293 cells, treated with PNGase F; lane 6: PNGase F. The IL-4 derived

from bacterial expression in SDS-gel 1, lane 2 serves reference for molecular size of non-glycosylated IL-4. PNGase F treatment leads always to a reduction in molecular mass, for the single glycosylated wildtype IL-4 (harbors the native N-glcosylation site at Asn38) glycan removal is complete, for multiglycosylated IL-4 variants, i.e., IL4 F82 T28N, IL-4 F82D K61N or IL-4 F82D (Q20N T28N K61N) also partially deglycosylated species are observed. This is since deglycosylation using the PNGase F Glycan Cleavage Kit is done under non-denaturing conditions and N-glycan moieties, which are partially (structurally) shielded in the protein, have a low rate of hydrolysis.

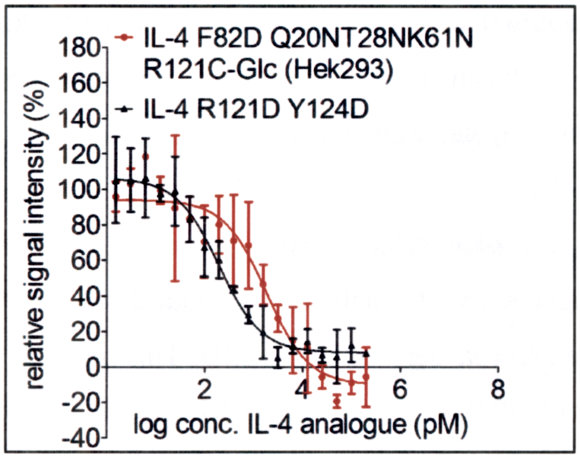

Figure S10: Dose-response curves of glycoengineered IL-4 variant F82D Q20N T28N K61N R121-Glc in TF1 cells. Cells were incubated with a log 2 titration of HEK293 cell-derived IL-4 F82D Q20N T28N K61N R121-Glc (protein was purified with Concanavalin A affinity chromatography) in competition with 50 pM wildtype IL-4 (*E. coli*-derived). The signal was normalized to wildtype IL-4 at 50 pM. For comparison the dose-response for *E. coli*-derived IL-4 R121D Y124D (Pitrakinra) in competition with 50 pM wildtype IL-4 is provided. Data from three independent experiments are shown.

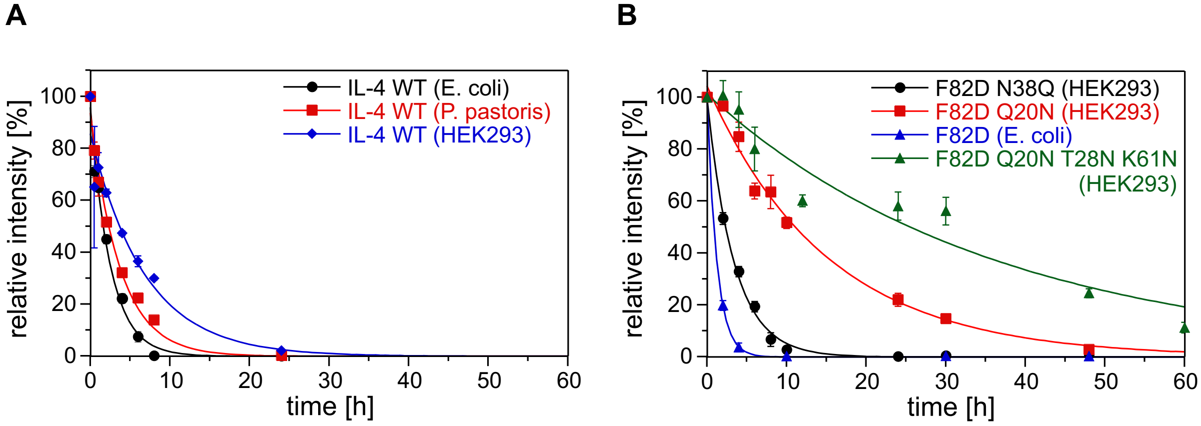

Figure S11: Analysis of time-dependent degradation of IL-4 proteins and glycoconjugates by trypsin. Wildtype IL-4 proteins derived from recombinant expression in either *E. coli*, *P. pastoris* or HEK293 cells were incubated with trypsin for 24 to 48 h (for experimental details see

materials and methods). Samples were taken at different time points and analyzed by SDS-PAGE analysis. Residual non-proteolyzed protein was quantified by gel densitometry using the software ImageLab (BioRad) and employing the volume tool. For relative quantification, staining intensities of the IL-4 protein bands were normalized to protein bands of the molecular weight standard. "Proteolytic half-lives" were determined using the software Prism applying the "one phase decay" fitting model. Alle experiments were performed as independent triplicates.

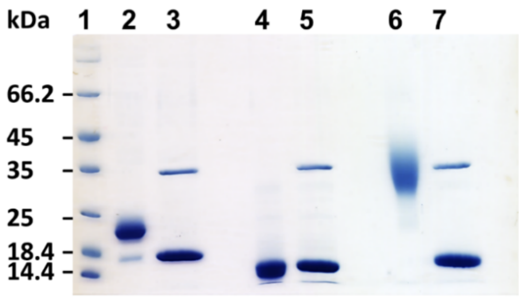

Figure S12: SDS-PAGE analysis of wildtype IL-4 from expression in *E. coli*, yeast and HEK293 cells. To determine the glycan-content, wildtype IL-4 proteins derived from different expression systems were deglycosylated using PNGase F and submitted to SDS-PAGE analysis together with non-treated samples. SDS-PAGE analysis was performed under reducing conditions. 1: Molecular weight standard; 2: IL-4 (HEK293); 3: IL-4 (HEK293) treated with PNGase F; 4: IL-4 (*E. coli*); 5: IL-4 (*E. coli*) treated with PNGase F; 6: IL-4 (*P. pastoris*); 7:IL-4 (*P. pastoris*) treated with PNGase F. The protein band observed at an apparent molecular weight of about 35 kDa in lanes 3, 5 and 7 is due to PNGase F added to the reaction mixture.

Additional references

1. Aricescu, A. R.; Lu, W. X.; Jones, E. Y., A time- and cost-efficient system for high-level protein production in mammalian cells. *Acta Crystallogr D* **2006,** *62*, 1243-1250.
2. Demeneix, B. A.; Boussif, O.; Zanta, M. A.; Remy, J. S.; Behr, J. P., Delivery of polynucleotides with polyamine lipids and polymers. *Nucleos Nucleot* **1997,** *16* (7-9), 1121-1127.
3. Richter, D.; Moraga, I.; Winkelmann, H.; Birkholz, O.; Wilmes, S.; Schulte, M.; Kraich, M.; Kenneweg, H.; Beutel, O.; Selenschik, P.; Paterok, D.; Gavutis, M.; Schmidt, T.; Garcia, K. C.; Muller, T. D.;

Piehler, J., Ligand-induced type II interleukin-4 receptor dimers are sustained by rapid re-association within plasma membrane microcompartments. *Nat Commun* **2017,** *8*, 15976.

4. Zacharius, R. M.; Zell, T. E.; Morrison, J. H.; Woodlock, J. J., Glycoprotein staining following electrophoresis on acrylamide gels. *Anal Biochem* **1969,** *30* (1), 148-52.
